# Emotional, Psychological, and Cognitive Changes Throughout the COVID-19 Pandemic in Italy: Is There an Advantage of Being an Older Adult?

**DOI:** 10.3389/fnagi.2021.712369

**Published:** 2021-09-10

**Authors:** Elena Carbone, Rocco Palumbo, Enrico Sella, Graziana Lenti, Alberto Di Domenico, Erika Borella

**Affiliations:** ^1^Department of General Psychology, University of Padova, Padua, Italy; ^2^Department of Psychological, Health and Territorial Sciences, G. d’Annunzio University of Chieti-Pescara, Chieti, Italy

**Keywords:** emotional functioning, psychological functioning, cognitive performance, age-related differences, COVID-19 pandemic

## Abstract

**Introduction:** The study examined age-related differences between young and older adults’ emotional and psychological experience as well as cognitive functioning throughout different phases of the COVID-19 pandemic in Italy.

**Materials and Methods:** Participants were interviewed by phone when confined at home during the national lockdown (T1-May 2020; *N* = 138 young adults; *N* = 119 older adults) and after the first wave of contagions, when restrictions were discarded (T2-September 2020; *N* = 52 young adults; *N* = 59 older adults). A sub-sample also participated in a third assessment (T3-December 2020). Participants completed questionnaires assessing their emotional and psychological functioning (i.e., positive and negative affect, perceived social and emotional loneliness, resilience) along with memory tasks (Backward Digit Span task and words list recall).

**Results:** Although individuals reported less positive and more negative emotions during the lockdown than at T2, results showed that older adults displayed overall fewer negative emotions and greater resilience than young adults. The latter were those who reported feeling more emotionally lonely when compared to their older counterpart during the lockdown than afterward. Older adults’ advantage in emotional and psychological functioning was also confirmed 7 months after the national lockdown. Only age-related differences in favor of young adults for the memory tasks were found. The measures of interest were also susceptible to mood and/or concerns of COVID-19 effects.

**Discussion:** These findings further highlight the age-related advantage of older adults managing the emotional and psychological experience even when facing an unexpected, prolonged, and unpredictable, stressful life event such as the COVID-19 pandemic.

## Introduction

The COVID-19 pandemic has represented an unexpected and prolonged stressful situation, with the potential of impacting individuals’ emotional and psychological functioning. Hence, a growing number of studies are interested in understanding the emotional and psychological consequences of the COVID-19 outbreak in young and healthy older adults.

The studies exploring age differences between young and older adults’ emotional reactions experienced during the first acute phase of the pandemic (March-June 2020) found that older adults showed higher emotional well-being (Carstensen et al., 2020), lower stress, and negative affect (Ceccato et al., 2020; Young et al., 2021) than younger adults when facing the unexpected and inescapable stressors imposed by the COVID-19 lockdown. During the lockdown, older individuals also felt less lonely than younger adults, thereby showing less vulnerability to the effects of social distancing (e.g., Bu et al., 2020; Luchetti et al., 2020; Varga et al., 2021). Furthermore, when considering resilience, a dynamic process of recovering from adversity which it configures as a crucial (psychological) protective factor to cope with the COVID-19 pandemic implications (e.g., Holmes et al., 2020), older adults’ perceived ability to cope with adversities was less influenced by COVID-19-related stressful events compared to younger adults (e.g., McCleskey and Gruda, 2021; Rossi et al., 2021). Such a pattern of findings showed how aging seemed to be associated with better emotional and psychological outcomes during the COVID-19 lockdown, which is in line with a well-proven theoretical framework on aging and emotions such as the Socioemotional Selectivity Theory (SST; Carstensen et al., 2003). According to the SST, due to their constrained temporal horizons, older adults experience motivational shifts toward prioritizing emotionally meaningful and positive goals and experiences with advancing age (e.g., Lockenhoff and Carstensen, 2004; Charles and Carstensen, 2010). Empirical evidence has, for instance, demonstrated that older adults display selective attention toward positive stimuli and show aversiveness toward negative ones (e.g., Fairfield et al., 2015). As a result, older adults experience high level of psychological well-being and display greater emotional regulation than young adults (Charles and Carstensen, 2010; Burr et al., 2021). Such a positive emotions-oriented attitude characteristic of older age may have therefore allowed older adults to display greater emotional and psychological functioning, than their younger counterparts, even when facing a stressful emergency such as the COVID-19 pandemic.

It is worth highlighting that these studies depicted the unique effects of the first wave of the COVID-19 outbreak (when individuals were confined at home due to the lockdown restrictions) and lack an “as-usual” everyday normal condition to compare data with. Moreover, the health emergency lasted throughout 2020. Like other life-stressful events, COVID-19 might have caused detrimental effects on individuals’ psychological and emotional well-being in the long term (Wang et al., 2020). Therefore, it is worth investigating to what extent the emotional and psychological effects of the pandemic and the age advantage, shown in such domains by older adults, change –or is maintained– over time.

Apart from young and older adults’ psychological and emotional fallout, which the SST can account, lockdown restrictions have also led individuals to cope with an impoverished environment, in terms of cognitive and socially stimulating activities and experiences, that are, however, known to have a role in counteracting/delaying age-related cognitive decline (Reuter-Lorenz and Park, 2014). At the same time, cognitive functioning is known also to be affected when stressful situations occur (e.g., Scott et al., 2015; Boals and Banks, 2020). Therefore, the lack of an “enriched” and stimulating environment, due to lockdown restrictions, and the unexpected and prolonged stressful emergency individuals had to manage, might have likely broadly impacted cognitive functioning as well. Interestingly, little attention has been devoted to understanding whether and to what extent the COVID-19 lockdown and the following restrictions have affected such other crucial domain of our functioning, as the cognitive one, however. The two studies that examined the COVID-19 lockdown influence in this domain, highlighted that attention, executive functions, and temporal orientation (Fiorenzato et al., 2021), as well as daily functioning (Reading Turchioe et al., 2021), were perceived as the most affected cognitive abilities during the lockdown, whereas an improvement in the memory domain was perceived (Fiorenzato et al., 2021). Also, younger adults reported higher cognitive complaints than their older counterpart (Fiorenzato et al., 2021; Reading Turchioe et al., 2021). These studies, however, focused on the very first wave of the pandemic and were based on self-reported measures that assessed participants’ perception of changes in their cognitive abilities during the lockdown compared to before. Thus, it is still unknown whether objective cognitive outcomes might have changed in young and older adults through the pandemic.

For the first time (at least to our knowledge), the present study aimed to ascertain age-related differences between young and older adults’ emotional and psychological experiences throughout different phases of the COVID-19 health emergency in Italy. In addition, changes in cognitive functioning, in particular in the memory domain, were jointly investigated using, for the first time, classical tasks assessing two memory domains sensitive to age-related decline, i.e., working memory -WM- (Borella et al., 2007, 2019; Gamboz et al., 2009) and episodic memory (Park, 2002).

To these aims, young and community-dwelling healthy older participants completed several questionnaires assessing different emotional and psychological factors (positive and negative affects, social and emotional loneliness, resilience), along with WM and long-term memory tasks (Backward Digit Span task and words list recall, respectively) on two occasions: (i) during the first national lockdown, when several activities (e.g., shops selling non-essential goods, educational –schools, university–, social and leisure activities services and infrastructures) were closed and people were allowed to leave their homes only for a short amount of time and for documented purposes (e.g., health issues, shopping for basic necessities, to go to work if this cannot be done from home) (T1; May 2020); and (ii) in the period following the first wave of the pandemic, when the stay-at-home and travel ban rules were dismissed and people were allowed again to engage work, social and leisure activities as usual (T2; September 2020). These two timepoints allowed us to depict age-related differences in the emotional and psychological experience and memory outcomes throughout the pandemic due to changes in restriction regulations. T2 indeed also served as an “as-usual” lifestyle routine situation condition (no restrictions) to be compared with the lockdown timepoint (T1). A third interview was also planned 7 months after the first lockdown (T3; December 2020), to follow up on the impact of the COVID-19 outbreak on psychological, emotional, and cognitive outcomes. What appeared to be the end of the emergency in our country in September (T2), however, was then followed by a second wave of contagions (since October 2020) that made it necessary to restore stricter restrictions rules encompassing national curfew, travel restrictions, and limited work, social and leisure activities. Our third assessment occasion (T3) was therefore unexpectedly characterized again by restrictions, thereby configuring as a condition that mirrored the one that occurred during the first timepoint (the national lockdown) more than an “as-usual” post-pandemic situation.

In line with previous studies (e.g., Carstensen et al., 2020), we expected older adults to show an advantage in terms of emotional and psychological outcomes during the different phases of the COVID-19 pandemic. Specifically, we expected older adults to (i) feel more positive and less negative emotions compared to younger adults (e.g., Luchetti et al., 2020); and (ii) be more resilient and less lonely than younger adults throughout the different timepoints (e.g., Rossi et al., 2021). Regarding the WM and episodic memory tasks, we explored whether any changes occurred between the two considered timepoints. Since the lockdown has impacted the everyday life cognitive and social “enriched” environment, we expected an accentuation of the age-related differences in favor of younger adults (e.g., Borella et al., 2007, 2019). However, given the short period (about 4 months) between the two timepoints, no cognitive changes due to the different restriction regulations were expected. Therefore, we finally explored whether changes in the examined measures were maintained or emerged in the longer term (T3). Since mood (depression) and worries of contagion from COVID-19 have been suggested to be among the vulnerability factors on mental health and cognitive outcomes associated with this stressful situation (e.g., Carstensen et al., 2020; De Pue et al., 2021), their impact on the psychological and emotional as well as cognitive outcomes considered was examined.

## Materials and Methods

### Participants

One hundred and forty-one young adults (age range: 18–44 years) and 157 older adults (age range: 64–82 years), all Italian and recruited by word of mouth, volunteered for the study and completed an individual phone interview during the national lockdown (T1; May 2020). Participants were contacted again in a subsequent period, and 52 young adults and 69 older adults agreed to participate in a second interview (T2; September 2020). Twenty-four young adults and 37 older adults also completed a third interview -which corresponded to a second wave of the pandemic– (T3; December 2020).

Exclusion criteria were: (i) current or past COVID-19 infection, (ii) serious health issues and/or use of medication (antidepressants, anxiolytics), as assessed by a semi-structured interview (De Beni et al., 2008), and (iii) for older adults, signs of cognitive impairment, i.e., a score below 17 to the Montreal Cognitive Assessment-BLIND classical and widely used cognitive functioning screening measure (Wittich et al., 2010).

The final sample included 138 young adults and 119 older adults at T1, 52 young adults and 59 older adults at T2, and 18 young adults and 31 older adults at T3.

### Materials

#### Emotional and Psychological Functioning

*The Positive and Negative Affect Schedule* (PANAS; Watson et al., 1988) consists of 20 adjectives describing different feelings assessing positive and negative affective states. Participants were asked to rate how they felt from 1 (not at all) to 5 (extremely) in the previous month (T1: April, T2: August, T3: November), higher scores corresponding to greater positive and negative affects, respectively (maximum = 50).

*The Social and Emotional Loneliness Scale* (Capotosto et al., 2017) comprises six items assessing emotional and social loneliness. Participants were asked to rate their agreement with each item from 1 (absolutely true) to 5 (absolutely not true) regarding the previous month (T1: April, T2: August, T3: November). The dependent variables were the sum of the three items for emotional and social loneliness, respectively, with lower scores indicating higher social and emotional loneliness (maximum = 15).

*The Connor-Davidson Resilience Scale–10 items* (Campbell-Sills and Stein, 2007) assesses the ability to cope with adversity. Participants were asked to rate each item from 0 (not true at all) to 4 (true nearly all the time) in relation to the previous month (T1: April, T2: August, T3: November), with higher scores corresponding to a greater resilience (maximum = 40).

#### Memory Performance

*The Backward Digit Span task* (adapted from the battery by De Beni et al., 2008) involves presenting a series of digits (1 s per digit). Participants had to repeat the series of digits in the backward order. The series started from two digits and rose to eight, each level containing two strings of digits. One point was assigned for each sequence correctly recalled. The final score corresponded to the total number of correct trials recalled (maximum = 14).

*The word list recall task* involves presenting an *ad hoc* list of 15 words (adapted from Carretti et al., 2011) at a rate of 2 s per word. Participants had to repeat as many words as they could remember immediately after the list was presented (immediate recall) and after 10 min (delayed recall). One point was assigned for each word correctly recalled. The final scores corresponded to the total number of correct words recalled for both the immediate and delayed recall tasks (maximum = 15), respectively.

Three parallel versions for each memory measure were created and used in a counterbalanced fashion across timepoints.

#### Mood and Fear of COVID-19

The *Beck Depression Inventory-II* (BDI-II; Beck et al., 1996) and the *Geriatric Depression Scale- 15 items* (GDS; Yesavage et al., 1982) are self-report of depressive symptoms for young and older adults, respectively.

The *Fear of COVID-19 questionnaire* (Di Crosta et al., 2020) assesses the conviction of being infected by COVID-19, either in the past or in the future (Beliefs of Contagion Scale -BCS), and the possibility of suffering severe consequences (i.e., being hospitalized or dying) due to the contagion (Consequences of Contagion Scale -CCS), referred to either self or loved ones’ health. Participants answered on a scale from 0 (not at all) to 100 (extremely) in relation to the previous month (T1: April, T2: August, T3: November), with higher scores corresponding to greater worries of being infected or suffering severe consequences due to the contagion, respectively.

### Procedure

Participants were contacted by phone to complete a single 90 min interview while confined at home due to the COVID-19 outbreak (T1; 1–20 May 2020). Participants were asked to place in a quiet area of their home to avoid hearing issues. After obtaining their consent, the experimenter guided participants through the completion of tasks and questionnaires -ensuring that they were able to hear and understand the instructions and stimuli clearly- as following: a semi-structured interview assessing demographic characteristics as well as physical and mental health status, the MOCA-BLIND (cognitive functioning screening for older adults only), the Backward Digit Span task, the word list-immediate recall, the Resilience scale, the word list-delayed recall, the Social and Emotional Loneliness Scale, the PANAS, the BDI-II (young adults) or the GDS (older adults), and the Fear of COVID-19 questionnaire. Participants completed the same survey also during the second (T2; 1–20 September 2020) and the third (T3; 1–20 December 2020) interviews.

### Statistical Analyses

To assess age-related differences between young and older adults’ mood (depression), worries of contagion from COVID-19, psychological and emotional experience, and memory performance throughout different phases of the COVID-19 pandemic, linear mixed-effects models (Pinheiro and Bates, 2000) were run for all the measures of interest with Age group (young adults vs. older adults) and Time (T1 vs. T2) as predictors, and random intercepts for participants. Maximum likelihood estimation was used. The significance of the effects was tested with the likelihood ratio test for nested models based on chi square distribution. Then, the psychological, emotional, and memory outcomes models were run again with mood (depression scores)^1^, BCS, and CCS as covariates to control for such vulnerability factors.

The same analyses (linear mixed-effects models with covariates) were also run considering the third interview (T3) to ascertain whether the pattern of results between T1 and T2 was confirmed. However, due to the small sample of participants that completed the third interview, these analyses have mainly descriptive/qualitative purposes.

## Results

The descriptive statistics of demographic characteristics, the measures of interest by age group and assessment occasion, and results from independent *t*-tests between young and older adults who did complete both interviews at T1 and at T2 and those who did complete only T1 are shown in Supplementary Materials-Section A.

### Mood and Fear of COVID-19

Regarding mood, a main effect of Time emerged for young adults only, who reported lower BDI-II scores at T2 than at T1 (see Supplementary Materials-Section B). The 28.26% of young adults and 15.96% of older adults at T1, and 7.69% of young adults and 15.25% of older adults at T2, scored above the cut-off of the BDI-II and the GDS, respectively.

As for the Fear of COVID-19 questionnaire, younger adults reported a higher belief of being infected by COVID-19 than older adults. In contrast, older adults reported greater worries of suffering severe consequences from contagion (see Supplementary Materials-Section B).

### Emotional and Psychological Functioning

Results showed only a significant main effect of Time for the PANAS-Positive emotions, participants reporting lower positive emotions at T1 than at T2. Significant main effects of Age group and Time emerged for the PANAS-Negative emotions. Older adults reported lower negative emotions than younger adults. Overall, participants reported higher negative emotions at T1 than at T2 (see Table 1).

**TABLE 1:** Results of the linear mixed-effects models for the measures of interest.

**Measure of interest**	**Effect**	**χ^2^**	**df**	***p***
PANAS-Positive emotions	Age group	1.620	1	0.203
	Time	24.872	1	<0.001
	Age group × Time	1.936	1	0.164
PANAS-Negative emotions	Age group	32.561	1	<0.001
	Time	13.174	1	<0.001
	Age group × Time	0.015	1	0.901
Emotional loneliness	Age group	7.352	1	0.007
	Time	41.139	1	<0.001
	Age group × Time	9.982	1	0.002
Social loneliness	Age group	0.987	1	0.321
	Time	3.058	1	0.080
	Age group × Time	1.206	1	0.272
Resilience	Age group	16.829	1	<0.001
	Time	0.138	1	0.710
	Age group × Time	0.031	1	0.860
Backward Digit Span task	Age group	40.748	1	<0.001
	Time	0.448	1	0.503
	Age group × Time	0.546	1	0.460
Word list-immediate recall	Age group	19.464	1	<0.001
	Time	4.396	1	0.036
	Age group × Time	2.171	1	0.141
Word list-delayed recall	Age group	20.160	1	<0.001
	Time	4.525	1	0.033
	Age group × Time	1.319	1	0.251

For the Emotional loneliness scale, significant main effects of Age group and Time emerged. Older adults reported lower emotional loneliness than younger adults. All participants reported higher emotional loneliness at T1 compared to T2 (see Table 1). The Age group × Time interaction was significant (see Table 1), and planned contrasts indicated that both young and older adults reported a higher emotional loneliness at T1 compared to T2 (ß = 1.94 [1.42; 2.46], *p* < 0.001; ß = 0.70 [0.19; 1.21], *p* = 0.032), and young adults perceived a higher emotional loneliness than older adults at T1 (ß = 1.36 [0.84; 1.88], *p* < 0.001), but not at T2 (ß = 0.12 [−0.62; 0.85], *p* = 1.00). For the Social loneliness scale, no significant effects emerged (see Table 1).

Regarding the Resilience scale, results showed only a significant main effect of Age group. Older adults reported higher resilience than younger adults (see Table 1).

**FIGURE 1:**
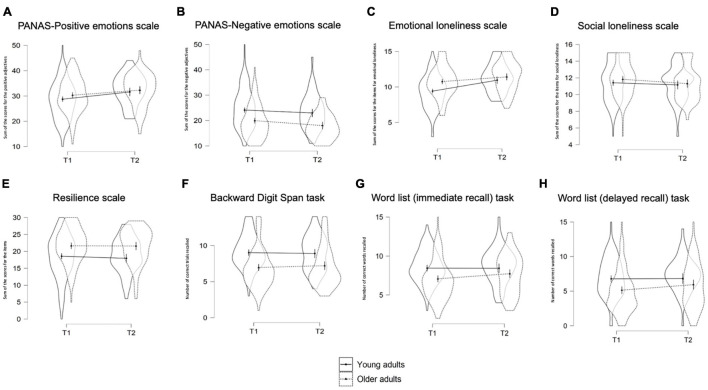
Models plots by age group (young adults vs. older adults) and time (T1-May 2020 vs. T2-September 2020) for each measure of interest. PANAS positive emotions scale **(A)**; PANAS negative emotions scale **(B)**; Emotional loneliness scale **(C)**; Social loneliness scale **(D)**; Resilience scale **(E)**; Backward Digit Span Task **(F)**; Word list immediate recall **(G)**; Word list delayed recall **(H)**. Plots of the models with mood, BCS, and CCS as covariates. For the Social and Emotional Loneliness scales lower scores correspond to higher perceived social and emotional loneliness, respectively.

When adding mood (depression), BCS, and CCS as covariates in the models, the mood was a significant covariate for all the emotional and psychological outcomes and the BCS was a significant covariate for the PANAS-Negative emotions (see Table 2 and Figure 1). Results were confirmed for all the outcomes except for the Social loneliness scale, for which a main effect of Time emerged. Individuals reported lower social loneliness at T1 compared to T2 (see Table 2).

**TABLE 2:** Results of the linear mixed-effects models including mood and beliefs and consequences of contagion scales as covariates.

**Measure of interest**	**Effect**	**χ^2^**	**df**	***p***
PANAS-positive emotions	Mood	76.594	1	<0.001
	Beliefs of contagion	0.550	1	0.458
	Consequences of contagion	0.445	1	0.505
	Age group	1.889	1	0.169
	Time	17.042	1	<0.001
	Age group × Time	0.566	1	0.452
PANAS-negative emotions	Mood	84.676	1	<0.001
	Beliefs of contagion	4.539	1	0.033
	Consequences of contagion	0.145	1	0.703
	Age group	30.135	1	<0.001
	Time	7.843	1	0.005
	Age group × Time	0.562	1	0.453
Emotional loneliness	Mood	90.586	1	<0.001
	Beliefs of contagion	3.126	1	0.077
	Consequences of contagion	0.004	1	0.949
	Age group	11.990	1	<0.001
	Time	32.221	1	<0.001
	Age group × Time	5.581	1	0.018
Social loneliness	Mood	16.328	1	<0.001
	Beliefs of contagion	1.928	1	0.165
	Consequences of contagion	2.482	1	0.115
	Age group	1.034	1	0.309
	Time	5.142	1	0.023
	Age group × Time	0.603	1	0.438
Resilience	Mood	69.851	1	<0.001
	Beliefs of contagion	2.663	1	0.103
	Consequences of contagion	0.152	1	0.697
	Age group	23.276	1	<0.001
	Time	0.629	1	0.428
	Age group × Time	0.419	1	0.518
Backward Digit Span task	Mood	1.626	1	0.202
	Beliefs of contagion	0.066	1	0.797
	Consequences of contagion	5.929	1	0.015
	Age group	28.712	1	<0.001
	Time	0.113	1	0.736
	Age group × Time	0.936	1	0.333
Word list-immediate recall	Mood	3.587	1	0.058
	Beliefs of contagion	3.672	1	0.055
	Consequences of contagion	1.568	1	0.210
	Age group	10.422	1	0.001
	Time	2.608	1	0.106
	Age group × Time	3.016	1	0.082
Word list-delayed recall	Mood	2.869	1	0.090
	Beliefs of contagion	6.561	1	0.010
	Consequences of contagion	2.032	1	0.154
	Age group	10.014	1	0.002
	Time	2.610	1	0.106
	Age group × Time	2.173	1	0.140

### Memory Performance

As for the Backward Digit Span task, results showed only a significant main effect of Age group with younger adults outperforming older adults (see Table 1).

Concerning the word list recall tasks, results showed a significant main effect of Age group for both the immediate and delayed recall with younger adults outperforming older adults. A significant main effect of Time also emerged for both the immediate and delayed recall. Participants recalled fewer words during the lockdown (T1) than at T2 (see Table 1).

When adding mood, the BCS, and the CCS as covariates in the model, the main effect of Time for both the immediate and delayed recall was no longer significant. The CCS scale was a significant covariate for the WM measure, and the BCS was a significant covariate for the word list delayed recall (see Table 2 and Figure 1).

### Additional Analyses

When considering T3 (see Supplementary Materials- Additional analyses section), older adults reported more positive and less negative emotions than younger adults for the PANAS scales. Regardless of age group, participants reported less positive and more negative emotions at T1 than T2, but no differences between T3 and T1 and T2 emerged.

Results on emotional loneliness confirmed lower scores for older adults than younger ones. Participants reported higher emotional loneliness at T1 than at T2 and felt less emotionally lonelier at T2 than at T3; the latter did not differ from T1. The Age group × Time interaction was no longer significant. For Social loneliness, the main effect of Time was no longer significant.

For the Resilience scale, the pattern of results found between T1 and T2 was confirmed. Older adults reported a higher resilience than their younger counterpart also in the long term.

Results at T3 also confirmed the age-related differences in favor of younger adults for the WM measure and the word list recall tasks.

The mood was always a significant covariate for the emotional and psychological outcomes and emerged as a significant covariate for the word list recall tasks. The BCS was always a significant covariate for the PANAS-Negative emotions and the word list delayed recall, while the CCS scale for the WM measure.

Finally, as a further additional analysis to investigate any associations between changes in emotional, psychological, and cognitive outcomes, correlations between change indexes expressing the difference between scores at T1 and scores at T2 for each measure of interest (controlled for mood, BCS, and CCS change indexes) were run by age group. Results showed that younger adults, who displayed better performance in the word list immediate recall task between the lockdown and the subsequent timepoint, showed greater positive affect between T1 and T2 (*r* = 0.31, *p* < 0.05). Furthermore, those who showed a better performance between T1 and T2 in the WM measure reported lower emotional loneliness (*r* = 0.31, *p* < 0.05). Instead, no significant associations between changes in the emotional, psychological, and cognitive outcomes emerged for older adults.

## Discussion

The present study examined age-related differences throughout the COVID-19 pandemic in Italy between young and older adults’ emotional and psychological experience and classical cognitive measures assessing memory abilities (not self-reported) important for everyday life functioning (WM and long-term memory) and sensitive to age-related effects. The lockdown phase (T1, restrictions), a subsequent timepoint mirroring an “as-usual” lifestyle routine situation (T2, no restrictions), and a third timepoint (T3, analyzed for qualitative purposes) were considered. The latter coincided with the second wave of the pandemic characterized by the reimplementation of the restrictions (a condition similar to T1).

In line with our expectations and previous evidence (e.g., Carstensen et al., 2020; Ceccato et al., 2020), our results showed that young and older adults displayed a different psychological and emotional experience of the COVID-19 lockdown. Although all individuals perceived higher negative and lower positive affect during the lockdown (T1) than in the subsequent “as-usual”-like situation (T2), older adults reported overall lower negative emotions, as well as a higher resilience (as discussed below), compared to their younger counterpart. The lockdown was also found to have a significant role on emotional loneliness, i.e., individuals reported negative feelings of detachment and lack of deep and meaningful relationships while confined at home (T1) more than in the subsequent timepoint (T2). This was particularly true for younger adults, who reported feeling emotionally lonelier than older adults, at T1 compared to T2. It is then worth highlighting that, in line with previous evidence (e.g., Rossi et al., 2021), compared with older adults, younger adults were those who reported higher depressive symptoms during the lockdown than afterward.

Such a pattern of findings for the emotional and psychological outcomes was also confirmed when considering T3 -albeit for qualitative purposes due to the small sample of young and older participants completing the third interview-. Older adults still showed better emotional and psychological functioning, together with higher positive emotions (not showed when considering only T1 and T2) than younger adults. However, at T3, participants’ scores on the Emotional loneliness scale tended to align with those reported at T1, and ratings on the PANAS scales were in between those reported at T1 and T2. Although T3 results need to be considered with caution, they further highlight the impact of restrictions imposing physical distancing on individuals’ emotional and psychological functioning and call upon the need to examine the effects of this emergency in the longer term.

It is worth mentioning that, though restrictions might be seen to have likely impacted young adults’ usual lifestyle habits the most, our older adult sample also reported to have a quite active lifestyle (i.e., engagement in several leisure, social and physical activities), and have had to cope with disrupted daily routines during the lockdown. Moreover, these results were found even though older adults reported greater worries of suffering severe consequences (i.e., being hospitalized or dying) due to the contagion than younger adults, but lower concerns about being infected by COVID-19, as suggested by the scores for the BCC and the CCS. Findings, therefore, are in line with the SST (Carstensen et al., 2003): the adaptive changes in emotion regulation that characterize older age (in which emotionally meaningful and positive goals and experiences are prioritized) might explain why, although both groups were worried about the COVID-19, older adults, compared to younger adults, were more able to regulate their emotional reactions, especially the negative affects also related to emotional loneliness. Furthermore, such a greater emotion regulation, along with several major life events faced throughout their life (and therefore a bigger wealth of experience), might also explain why older adults reported a higher resilience compared to younger adults (e.g., Rossi et al., 2021), thereby feeling able to face, cope, and adapt more easily than their younger counterparts to such an unexpected, unpredictable and stressful situation.

Intriguingly, when considering the perceived amount of social support, expressed by the social loneliness facet, young and older adults reported to perceive a higher lack of social support at T2 than at T1, a pattern that emerged when considering mood and concerns of COVID-19 effects and which disappeared when also T3 was considered. The enhanced sense of community (a shared feeling of “everyone being in it together”), emerged with the COVID-19 outbreak, together with the use of technology to maintain social contacts and interactions, might have led individuals to feel connected with their support network, especially even when physically isolated (e.g., Luchetti et al., 2020). We did not ascertain neither participants’ feeling of connectedness nor their social media usage, however, therefore these are only speculations that merit further investigations.

Regarding cognitive outcomes, our findings do not seem to align with previous evidence (Fiorenzato et al., 2021; Reading Turchioe et al., 2021), which included self-reported measures of cognitive complaints leading to an over or underestimation of cognitive changes compared to objective outcomes implemented in this study. The well-known age-related differences in favor of younger adults (e.g., Park, 2002; Borella et al., 2007) were confirmed for both the WM measure and the word list recall tasks. A different pattern of results emerged depending on the memory outcomes considered when the different timepoints effects were examined. Performance in the WM measure was not affected by restrictions, regardless of controlling or not for mood and COVID-19 concerns. Long-term memory outcomes, instead, showed an improvement between T1 and T2. However, these latter outcomes seemed to be particularly susceptible to the influence of mood (that emerged more clearly when also considering T3) and COVID-19 concerns, which overshadowed any timepoints effects between T1 and T2. Such a pattern of results suggests how, rather than lockdown restrictions *per se*, mood changes and worries related to stressful situations might impact memory outcomes based on the demands and the content of the tasks considered, particularly long-term memory ones considered here. Moreover, associations between positive changes in terms of emotional functioning and a better memory performance emerged for younger adults only. These results might suggest how the less adaptive coping and emotional responses displayed by younger adults influenced their memory performance, whereas the functional emotion regulation of older adults might have allowed them to maintain (not worsen) their memory performance. It is worth stressing that the relatively short period of time (about 4 months between T1 and T2 and about 6 months from T1 when considering T3) occurred between the considered timepoints, might have prevented to clearly observe the impact of the lockdown restrictions and the resulted “impoverished” environment, as well as any interplay between the psychological and emotional response to the emergency and cognitive functioning in young and older adults. Further investigations are therefore needed to ascertain whether the ongoing stressful situation might have a fallout on cognitive functioning in the longer term, also as a function of the different psychological and emotional responses displayed by young and older adults and the demands and content of the tasks in hand – e.g., considering memory tasks with emotional stimuli, as well as other cognitive functions, essential in everyday life (e.g., Meneghetti et al., 2011, 2014).

Despite these interesting findings, some limitations have to be acknowledged. A pre-pandemic baseline assessment would have helped to better depict the emotional, psychological, and cognitive fallout of such stressful and extraordinary times throughout the different pandemic phases (in terms of restrictions). Although one of our study’s strengths consists of having data not limited to the restrictions period *per se*, results on T3 should be considered with caution and for qualitative purposes only, due to the small sample of individuals completing the assessment. However, based on our results, and since the pandemic is still ongoing, it seems paramount to keep ascertaining the psychological, emotional, and cognitive fallout of such extraordinary times also as a function of the changes in the restrictions that lead to an unpredictable variation of lifestyle habits, and availability of cognitive stimulating experiences. In doing so, another aspect that should be considered is whether the spread of COVID-19 (in terms of number of contagions in different geographic areas) affected individuals’ emotional and psychological experience and reactions to the emergency differently. We attempted to control this aspect by administering the two Fear of COVID-19 scales, created to assess individuals’ concerns toward being infected by COVID-19 and suffering from severe consequences due to contagion. Moreover, other individual characteristics (e.g., personality dispositions) and protective or risk factors (i.e., coping styles), as well as other cognitive functions, not examined in this study, should be considered in the future as they might help delineate individuals at risk of suffering from negative consequences when this emergency will end.

Overall, our results suggest that older adults take advantage of their emotional strengths and resilience even when facing new, unexpected, and prolonged threats as the COVID-19 pandemic. However, findings also call upon the need to implement interventions that promote functional coping strategies to improve emotion regulation, resilience, and the quality of social relationships, especially for young adults, during unforeseeable and prolonged circumstances that impose physical distancing.

## Data Availability Statement

The raw data supporting the conclusions of this article will be made available by the authors, without undue reservation.

## Ethics Statement

The studies involving human participants were reviewed and approved by the Ethical Committee for the Psychological Research of the School of Psychology, University of Padova, Padua, Italy. The patients/participants provided their written informed consent to participate in this study.

## Author Contributions

EC contributed to supervising the data collection, analyzing and interpreting the data, and writing the manuscript. RP contributed to designing the study, interpreting the data, and writing the manuscript. ES supervised the data collection and drafted the manuscript. GL contributed to enrolling and interviewing participants, supervising the data collection, and organizing the database. ADD contributed to designing the study and drafted the manuscript. EB designed the study, contributed to analyzing, interpreting the data, and writing the manuscript. All authors read and approved the final manuscript.

## Conflict of Interest

The authors declare that the research was conducted in the absence of any commercial or financial relationships that could be construed as a potential conflict of interest. The reviewer MF declared a shared affiliation, though no other collaboration, with several of the authors RP and ADD to the handling editor.

## Publisher’s Note

All claims expressed in this article are solely those of the authors and do not necessarily represent those of their affiliated organizations, or those of the publisher, the editors and the reviewers. Any product that may be evaluated in this article, or claim that may be made by its manufacturer, is not guaranteed or endorsed by the publisher.

## Footnotes

1 To obtain a unique measure for depressive symptoms to be used as a covariate in the analyses, standardized scores, age group-centered considering the scores at T1, were computed.
