# Supplementary material for: Emotional, Psychological, and Cognitive Changes Throughout the COVID-19 Pandemic in Italy: Is There an Advantage of Being an Older Adult?
Source: Front Aging Neurosci. 2021 Sep 10;13:712369. doi: 10.3389/fnagi.2021.712369 (PMC8462933; doi:10.3389/fnagi.2021.712369)
Supplement: Supplementary file 1 [file Data_Sheet_1.docx]

**Supplementary materials**

**Emotional, psychological, and cognitive changes throughout the COVID-19 pandemic in Italy: Is there an advantage of being an older adult?**

**Summary**

**Section A-** Descriptive statistics and differences at T1 between participants who completed both the T1 and T2 assessments, and those who did not complete the second interview.

**Section B-** Age-related differences in mood and the Beliefs and Consequences of contagion scales.

**Additional analyses section**- Results from the linear mixed-effects models run considering the third assessment point (T3; December, 2020).

**Section A**

Table S1. Descriptive statistics of the demographic characteristics and the measures of interest by age group and assessment occasion.

|  | T1 (May 2020) | | | | T2 (September 2020) | | | | T3 (December 2020) | | | |
| --- | --- | --- | --- | --- | --- | --- | --- | --- | --- | --- | --- | --- |
|  | Younger Adults  (N=138; 78 F) | | Older Adults  (N=119; 79 F) | | Younger Adults  (N=52; 33 F) | | Older Adults  (N=59; 38 F) | | Younger Adults  (N=18; 12 F) | | Older Adults  (N=31; 17 F) | |
|  | M | SD | M | SD | M | SD | M | SD | M | SD | M | SD |
| Age | 24.59 | 4.27 | 69.83 | 5.60 | 24.83 | 4.75 | 69.86 | 4.67 | 24.28 | 4.17 | 69.06 | 3.57 |
| Education (years) | 15.78 | 2.34 | 11.56 | 4.34 | 15.81 | 2.36 | 11.59 | 4.08 | 16.33 | 2.43 | 12.35 | 3.07 |
| MOCA-Blind | - | - | 19.77 | 1.81 | - | - | 19.83 | 1.77 | - | - | 20.06 | 1.57 |
| Mood: |  |  |  |  |  |  |  |  |  |  |  |  |
| *Beck Depression Inventory-II* | 10.87 | 7.39 | - | - | 8.40 | 5.74 | - | - | 7.61 | 4.49 | - | - |
| *Geriatric Depression Scale* | - | - | 3.39 | 2.39 | - | - | 3.31 | 2.26 | - | - | 3.32 | 2.30 |
| Fear of COVID-19: |  |  |  |  |  |  |  |  |  |  |  |  |
| *Beliefs about contagion* | 37.39 | 21.68 | 23.06 | 17.47 | 40.83 | 20.31 | 24.18 | 16.04 | 55.76 | 17.99 | 31.80 | 18.12 |
| *Consequences of contagion* | 40.68 | 20.25 | 52.06 | 24.28 | 38.49 | 17.33 | 52.58 | 24.66 | 41.56 | 11.85 | 49.37 | 18.60 |
| Emotional and psychological  functioning: |  |  |  |  |  |  |  |  |  |  |  |  |
| *PANAS-Positive emotions* | 28.41 | 7.37 | 30.30 | 6.72 | 32.62 | 5.82 | 32.37 | 6.42 | 30.83 | 5.36 | 32.10 | 5.02 |
| *PANAS-Negative emotions* | 24.74 | 7.64 | 19.61 | 6.55 | 23.27 | 6.80 | 17.56 | 4.83 | 23.22 | 6.62 | 17.90 | 6.17 |
| *Emotional loneliness* | 9.33 | 2.32 | 10.70 | 2.12 | 11.23 | 1.91 | 11.46 | 2.05 | 10.28 | 2.47 | 10.58 | 1.79 |
| *Social loneliness* | 11.37 | 2.20 | 11.82 | 2.01 | 11.21 | 2.05 | 11.20 | 1.97 | 12.06 | 1.63 | 11.29 | 1.77 |
| *Resilience* | 18.41 | 6.25 | 21.45 | 5.18 | 18.87 | 5.02 | 21.56 | 5.15 | 19.83 | 5.50 | 20.19 | 4.53 |
| Memory performance: |  |  |  |  |  |  |  |  |  |  |  |  |
| *Backward Digit Span task* | 9.09 | 2.31 | 6.86 | 2.69 | 8.94 | 2.43 | 7.08 | 2.76 | 9.72 | 2.27 | 7.84 | 2.56 |
| *Word list (immediate recall)* | 8.54 | 2.07 | 6.95 | 2.50 | 8.75 | 2.22 | 7.71 | 2.51 | 8.89 | 2.87 | 8.19 | 3.12 |
| *Word list (delayed recall)* | 6.88 | 2.84 | 4.92 | 3.01 | 7.29 | 2.42 | 5.88 | 3.26 | 9.41 | 1.87 | 6.23 | 3.00 |

Note. MOCA-Blind: Montreal Cognitive Assessment-Blind; PANAS: Positive Affect and Negative Affect Scales.

Table S2. Differences at T1 for the measures of interest between younger adults who completed also the second interview and who did not complete the second interview.

|  | Younger adults who completed T1 and T2 (N=52) | | Younger adults who completed T1 only (N=86) | | Differences at T1 | | | | | |
| --- | --- | --- | --- | --- | --- | --- | --- | --- | --- | --- |
|  | M | SD | M | SD | t | gl | *p* | Cohen's d | lower CI 95% | upper CI 95% |
| Age | 24.83 | 4.75 | 24.45 | 3.97 | .50 | 136 | .62 | .09 | -.26 | .43 |
| Education (years) | 15.81 | 2.36 | 15.76 | 2.35 | .13 | 136 | .90 | .02 | -.32 | .37 |
| Beck Depression Inventory-II | 10.27 | 6.69 | 11.23 | 7.80 | -.74 | 136 | .46 | -.13 | -.48 | .22 |
| Fear of COVID-19: |  |  |  |  |  |  |  |  |  |  |
| *Beliefs about contagion* | 36.74 | 21.55 | 37.79 | 21.87 | -.27 | 135 | .78 | -.05 | -.39 | .30 |
| *Consequences of contagion* | 42.48 | 20.75 | 39.59 | 19.98 | .81 | 135 | .42 | .14 | -.20 | .49 |
| Emotional and psychological functioning: |  |  |  |  |  |  |  |  |  |  |
| *PANAS-Positive emotions* | 29.19 | 6.80 | 27.93 | 7.70 | .97 | 135 | .33 | .17 | -.17 | .51 |
| *PANAS-Negative emotions* | 25.90 | 7.15 | 24.04 | 7.87 | 1.39 | 135 | .16 | .24 | -.10 | .59 |
| *Emotional loneliness* | 9.23 | 1.90 | 9.40 | 2.55 | -.40 | 136 | .69 | -.07 | -.42 | .27 |
| *Social loneliness* | 11.29 | 2.12 | 11.42 | 2.26 | -.34 | 136 | .74 | -.06 | -.40 | .29 |
| *Resilience* | 18.75 | 5.27 | 18.20 | 6.80 | .50 | 136 | .62 | .09 | -.26 | .43 |
| Memory performance: |  |  |  |  |  |  |  |  |  |  |
| *Backward Digit Span task* | 8.88 | 2.48 | 9.21 | 2.21 | -.80 | 136 | .43 | -.14 | -.49 | .21 |
| *Word list (immediate recall)* | 8.69 | 1.92 | 8.45 | 2.18 | .63 | 127 | .52 | .11 | -.23 | .46 |
| *Word list (delayed recall)* | 7.19 | 2.14 | 6.69 | 3.19 | 1.02 | 136 | .31 | .18 | -.17 | .52 |

Table S3. Differences at T1 for the measures of interest between older adults who completed also the second interview and who did not complete the second interview.

|  | Older adults who completed T1 and T2 (N=59) | | Older adults who completed T1 only (N=60) | | Differences at T1 | | | | | |
| --- | --- | --- | --- | --- | --- | --- | --- | --- | --- | --- |
|  | M | SD | M | SD | t | gl | *p* | Cohen's d | lower CI 95% | upper CI 95% |
| Age | 69.86 | 4.67 | 69.80 | 6.43 | .06 | 117 | .95 | .01 | -.35 | .37 |
| Education (years) | 11.59 | 4.08 | 11.53 | 4.62 | .08 | 117 | .94 | .01 | -.34 | .37 |
| MOCA-Blind | 19.78 | 1.64 | 19.77 | 1.98 | .04 | 117 | .97 | .01 | -.35 | .36 |
| Geriatric Depression Scale | 3.42 | 2.61 | 3.35 | 2.19 | .17 | 117 | .87 | .03 | -.33 | .39 |
| Fear of COVID-19: |  |  |  |  |  |  |  |  |  |  |
| *Beliefs about contagion* | 22.00 | 19.80 | 24.10 | 14.92 | -.65 | 117 | .51 | -.12 | -.48 | .24 |
| *Consequences of contagion* | 53.64 | 24.61 | 50.51 | 24.06 | .70 | 117 | .48 | .13 | -.23 | .49 |
| Emotional and psychological unctioning: |  |  |  |  |  |  |  |  |  |  |
| *PANAS-Positive emotions* | 30.12 | 6.61 | 30.48 | 6.89 | -.30 | 117 | .77 | -.05 | -.41 | .31 |
| *PANAS-Negative emotions* | 19.56 | 6.59 | 19.65 | 6.56 | -.08 | 117 | .94 | -.01 | -.37 | .35 |
| *Emotional loneliness* | 10.81 | 2.05 | 10.58 | 2.20 | .59 | 117 | .56 | .11 | -.25 | .47 |
| *Social loneliness* | 11.59 | 1.93 | 12.03 | 2.08 | -1.20 | 117 | .23 | -.22 | -.58 | .14 |
| *Resilience* | 21.49 | 4.84 | 21.42 | 5.53 | .08 | 117 | .94 | .01 | -.35 | .37 |
| Memory performance: |  |  |  |  |  |  |  |  |  |  |
| *Backward Digit Span Task* | 6.80 | 2.55 | 6.92 | 2.85 | -.24 | 117 | .81 | -.04 | -.40 | .32 |
| *Word list (immediate recall)* | 7.08 | 2.67 | 6.82 | 2.34 | .58 | 117 | .56 | .11 | -.25 | .47 |
| *Word list (delayed recall)* | 5.17 | 3.05 | 4.67 | 2.98 | .91 | 117 | .37 | .17 | -.19 | .53 |

Note. MOCA-Blind: Montreal Cognitive Assessment-Blind; PANAS: Positive Affect and Negative Affect Scales.

**Section B**

Linear mixed-effects models (Pinheiro and Bates, 2000) were run for mood (depression) and the Beliefs and Consequences of contagion scales with Age group (young adults vs. older adults) and Time (T1 vs. T2) as predictors, and random intercepts for participants. Maximum likelihood estimation was used with likelihood ratio test to assess the significance of the effects.

**Mood (depression scores)**

*Figure S1. Model plots for the Beck Depression Inventory-II (young adults) and the Geriatric Depression Scale (older adults) scores by time.*


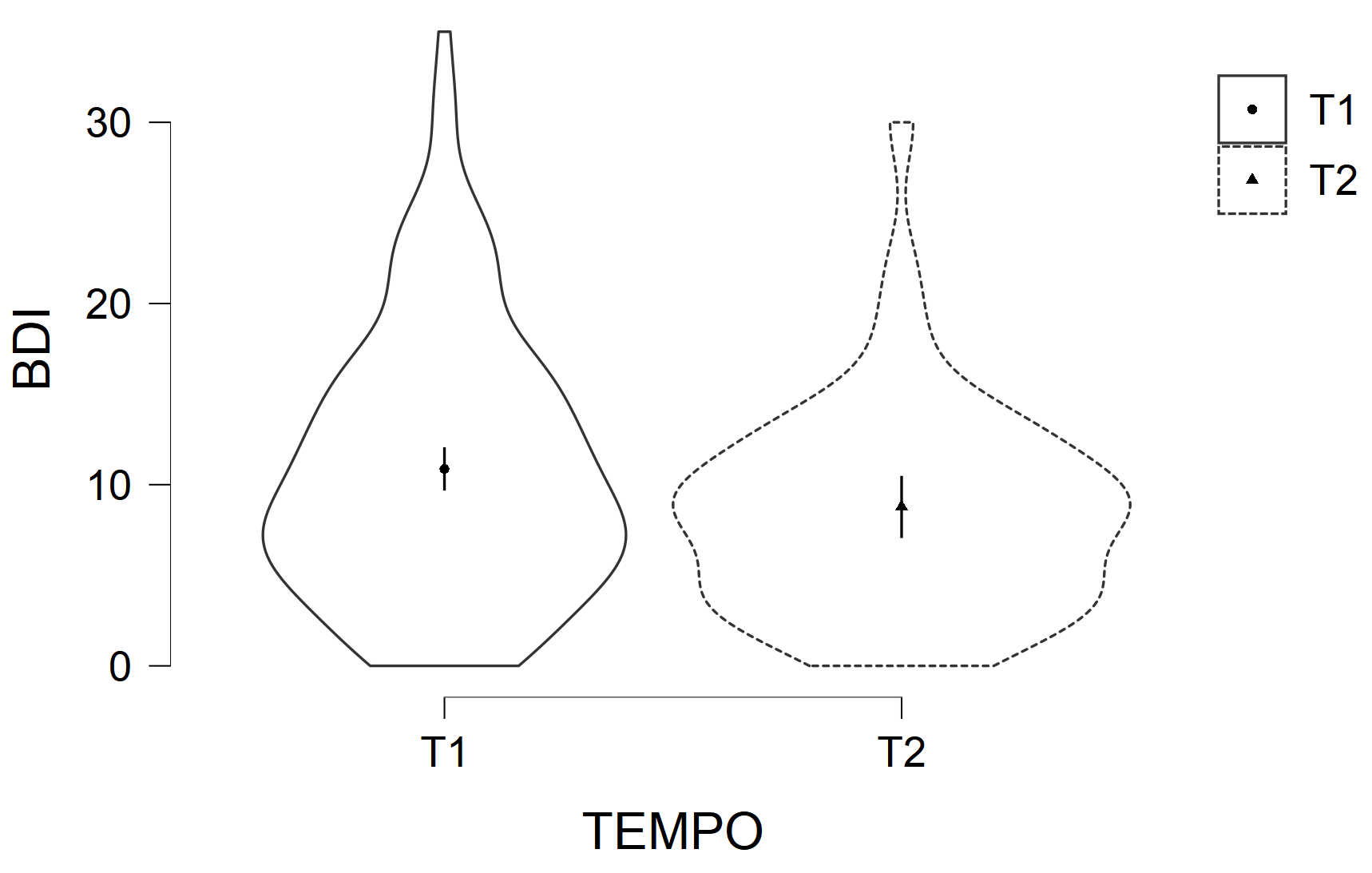


Geriatric Depression Scale (older adults)

Beck Depression Inventory-II (young adults)


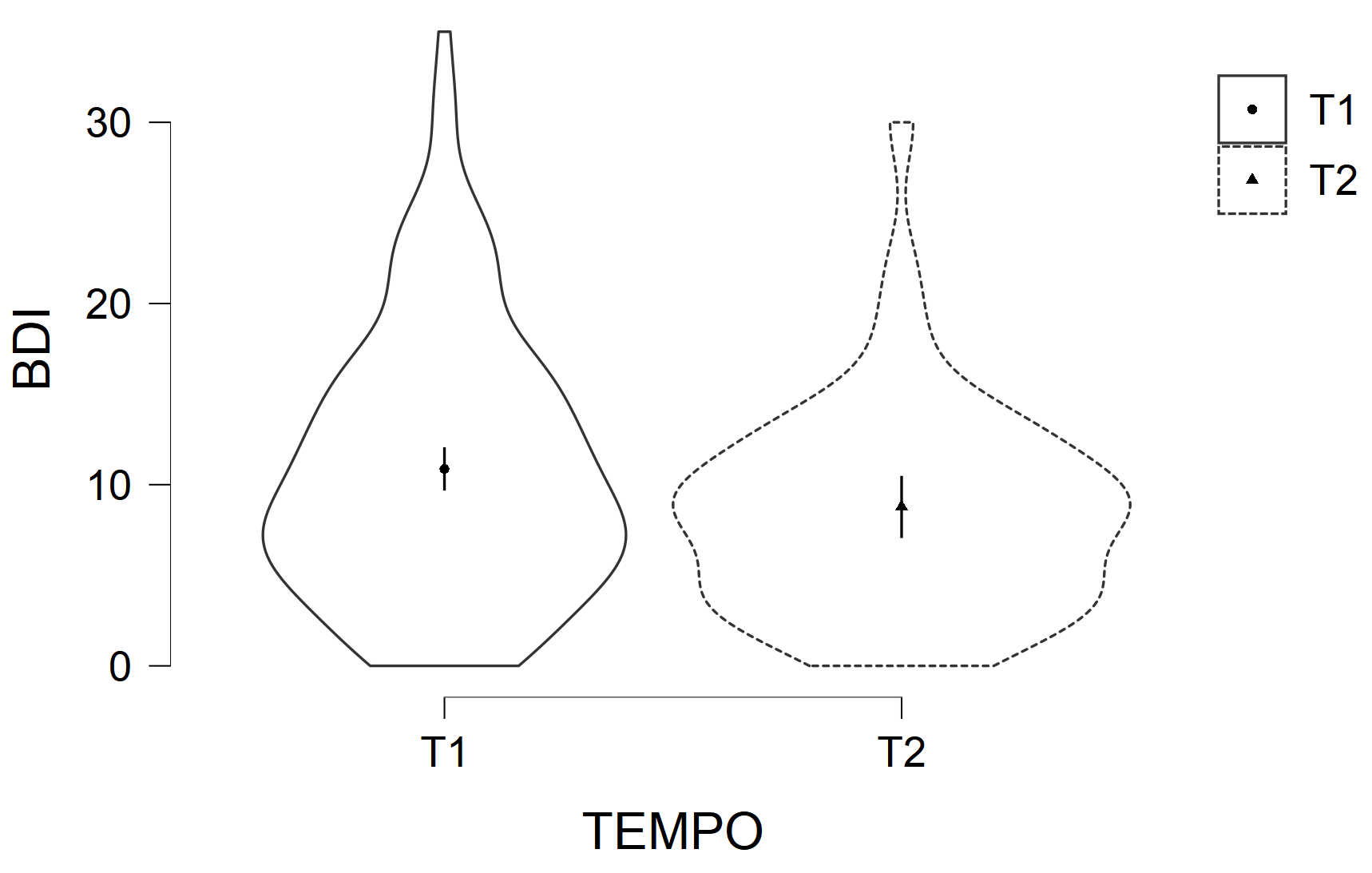

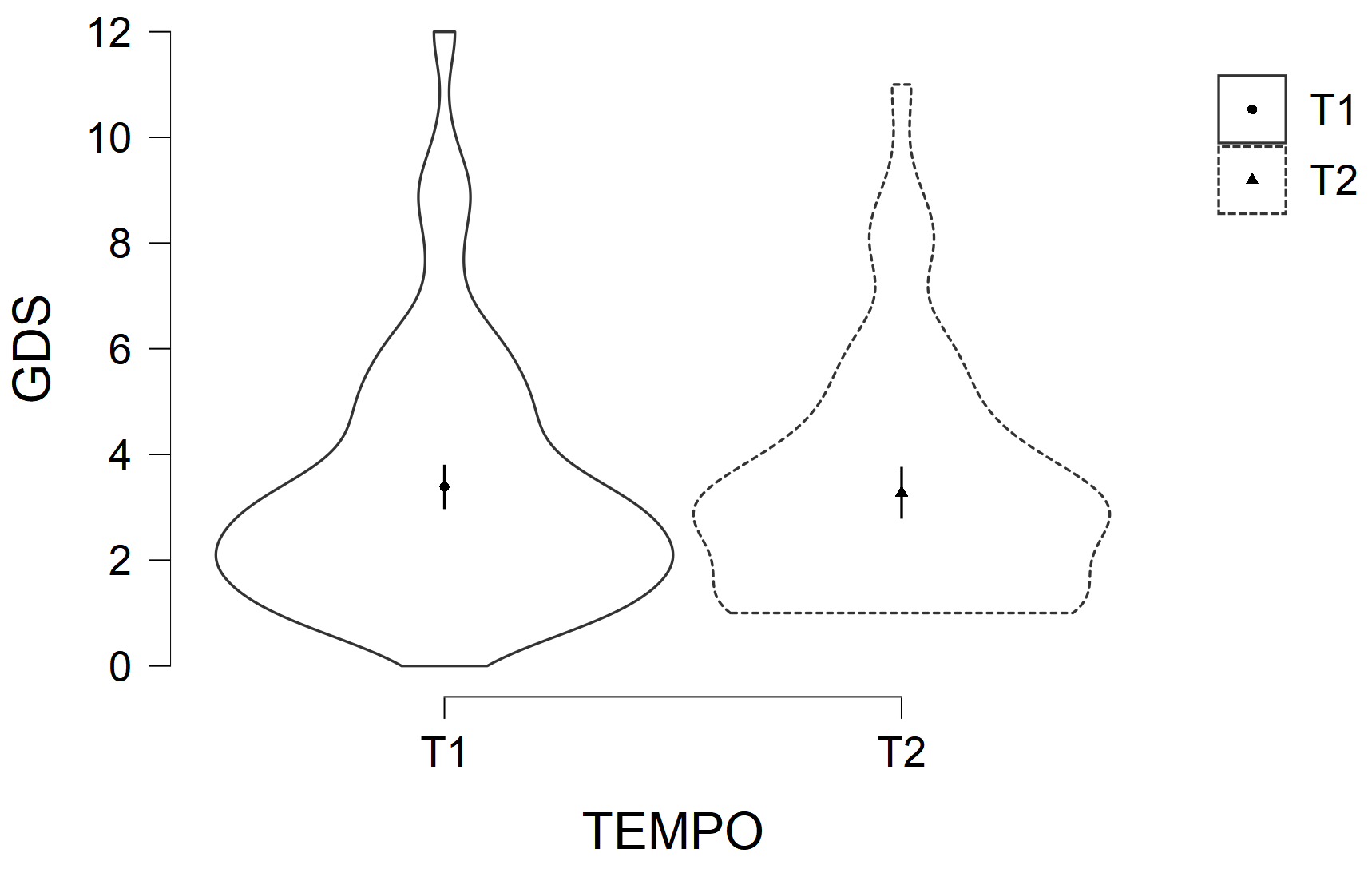


*Table S4. Models’ summary.*

|  | Beck Depression Inventory-II  (young adults) | | | Geriatric Depression Scale  (older adults) | | |
| --- | --- | --- | --- | --- | --- | --- |
| **Effect** | **df** | **χ^2^** | ***p*** | **df** | **χ^2^** | ***p*** |
| Time | 1 | 6.500 | .011 | 1 | .369 | .544 |

Results showed a main effect of Time for young adults, but not for older adults. Young adults reported higher BDI-II scores at T1 than at T2 (ß = -2.10 [-3.68; -0.51] *p* = .010).

When considering also the third assessment occasion (T3; December 2020), this pattern of results was confirmed.

**Beliefs of Contagion scale**

*Figure S2. Model plot for the Beliefs of Contagion scale by age group and time.*


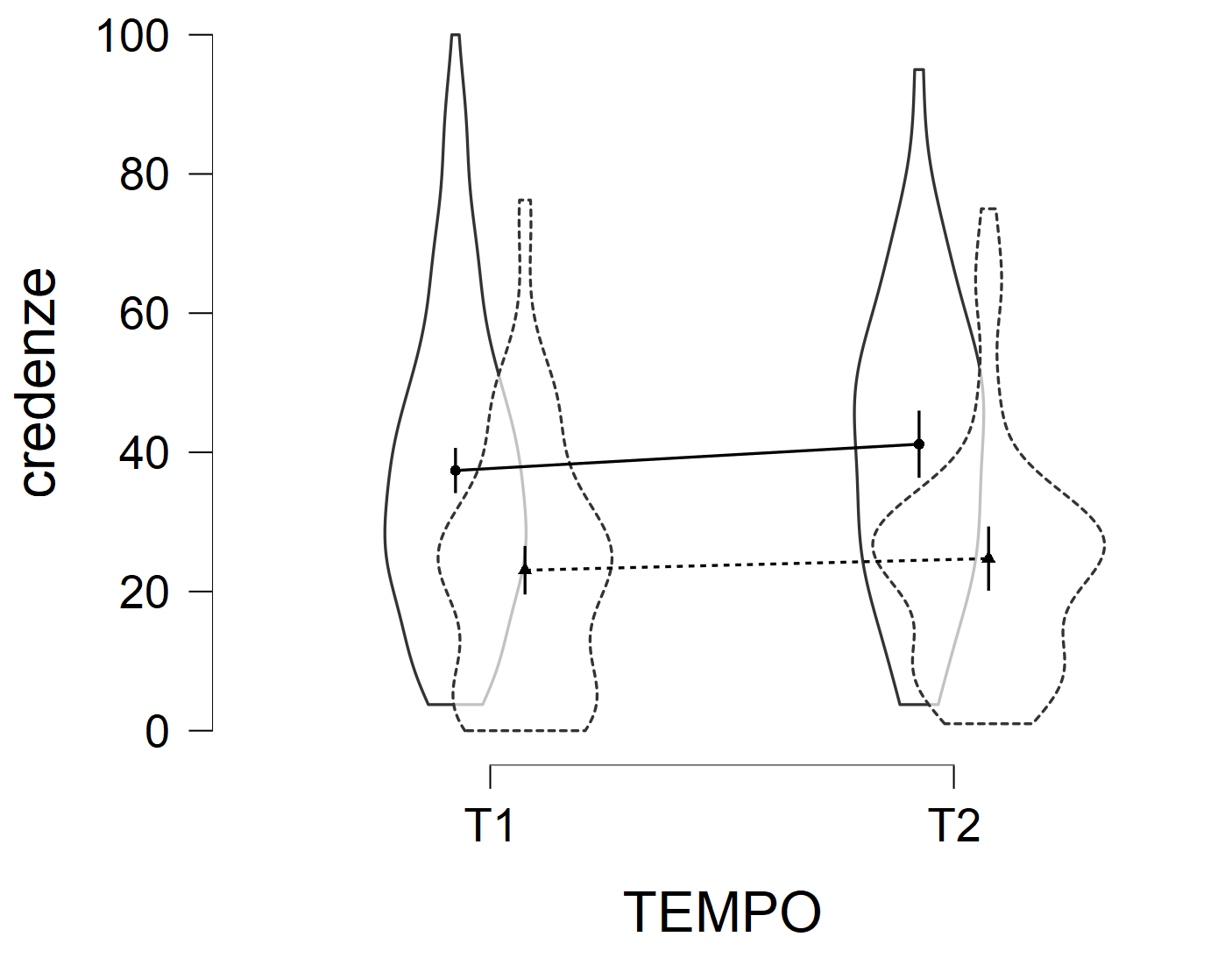

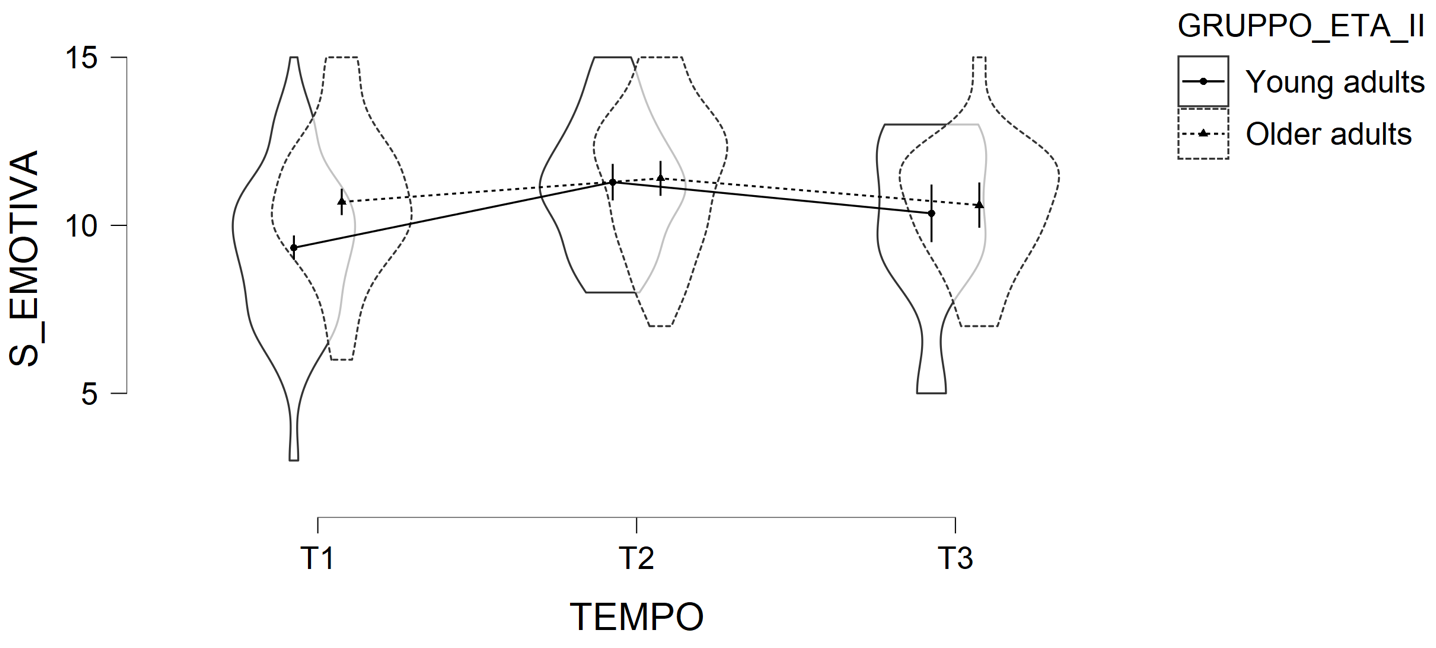


*Table S5. Model summary.*

| **Effect** | | **df** | | **χ^2^** | | ***p*** | |
| --- | --- | --- | --- | --- | --- | --- | --- |
| Age group |  | 1 |  | 38.115 |  | < .001 |  |
| Time |  | 1 |  | 2.644 |  | .104 |  |
| Age group X Time |  | 1 |  | 0.399 |  | .528 |  |

Results showed a main effect of Age group. Younger adults reported higher beliefs for themselves or loved ones of being infected by COVID-19 than older adults (ß = 15.38 [10.63; 20.33] *p* < .001).

When considering also the third assessment occasion (T3; December 2020), a main effect of Time also emerged (χ^2^(2) = 30.29, *p* <.001). Participants reported higher beliefs of being infected by COVID-19 between T2 and T3 (ß = 10.57 [5.85; 15.24] *p* < .001), and at T3 than at T1 (ß = 13.33 [8.77; 17.89] *p* < .001).

**Consequences of Contagion scale**

*Figure S3. Model plot for the Consequences of Contagion scale by age group and time.*


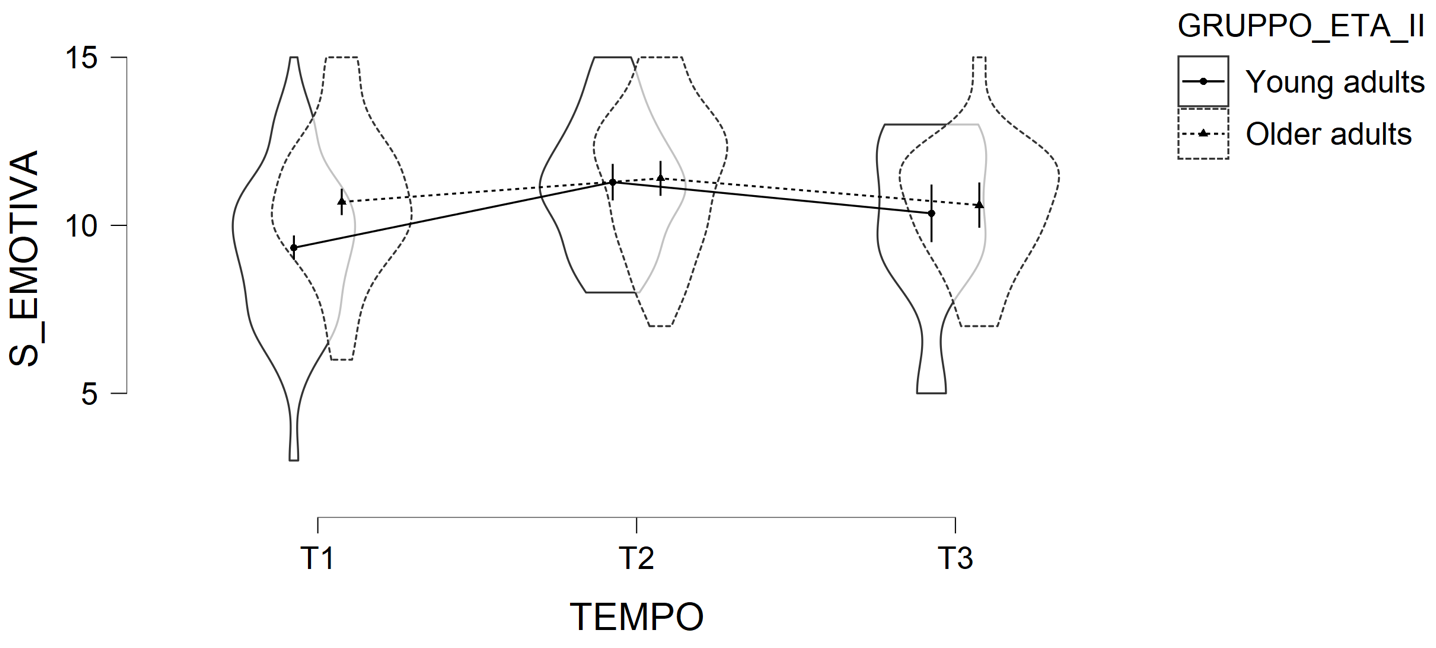

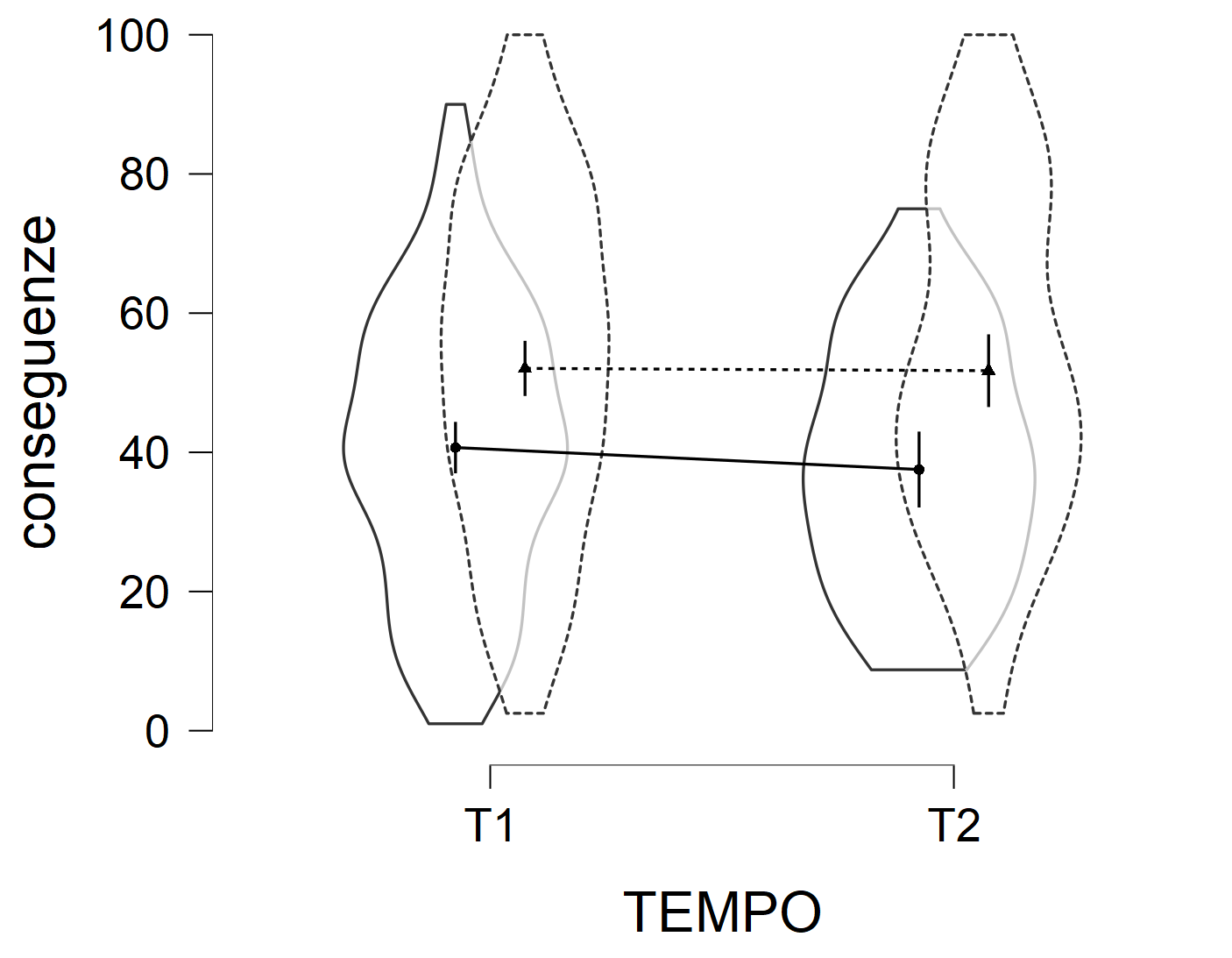


*Table S6. Model summary.*

| **Effect** | | **df** | | **χ^2^** | | ***p*** | |
| --- | --- | --- | --- | --- | --- | --- | --- |
| Age group |  | 1 |  | 20.988 |  | < .001 |  |
| Time |  | 1 |  | .860 |  | .354 |  |
| Age group X Time |  | 1 |  | .572 |  | .449 |  |

Results showed a main effect of Age group. Older adults reported higher beliefs for themselves or loved ones of suffering severe consequences (i.e., being hospitalized or dying) due to the contagion than younger adults (ß = 12.79 [7.42; 18.16] *p* < .001).

When considering also the third assessment occasion (T3; December 2020) this pattern of result was confirmed.

**Additional analyses section**

**Psychological and emotional functioning**

Linear mixed-effects models were run for all the emotional and psychological measures, with Age group (young adults vs. older adults) and Time (T1 vs. T2 vs. T3) as predictors, mood (depression) and the Beliefs and Consequences of contagion scales -BCS and CCS- as covariates. Maximum likelihood estimation was used with likelihood ratio test to assess the significance of the effects.

***PANAS-Positive emotions***

*Figure S4. Model plot for the PANAS-Positive emotions by age group and time.*


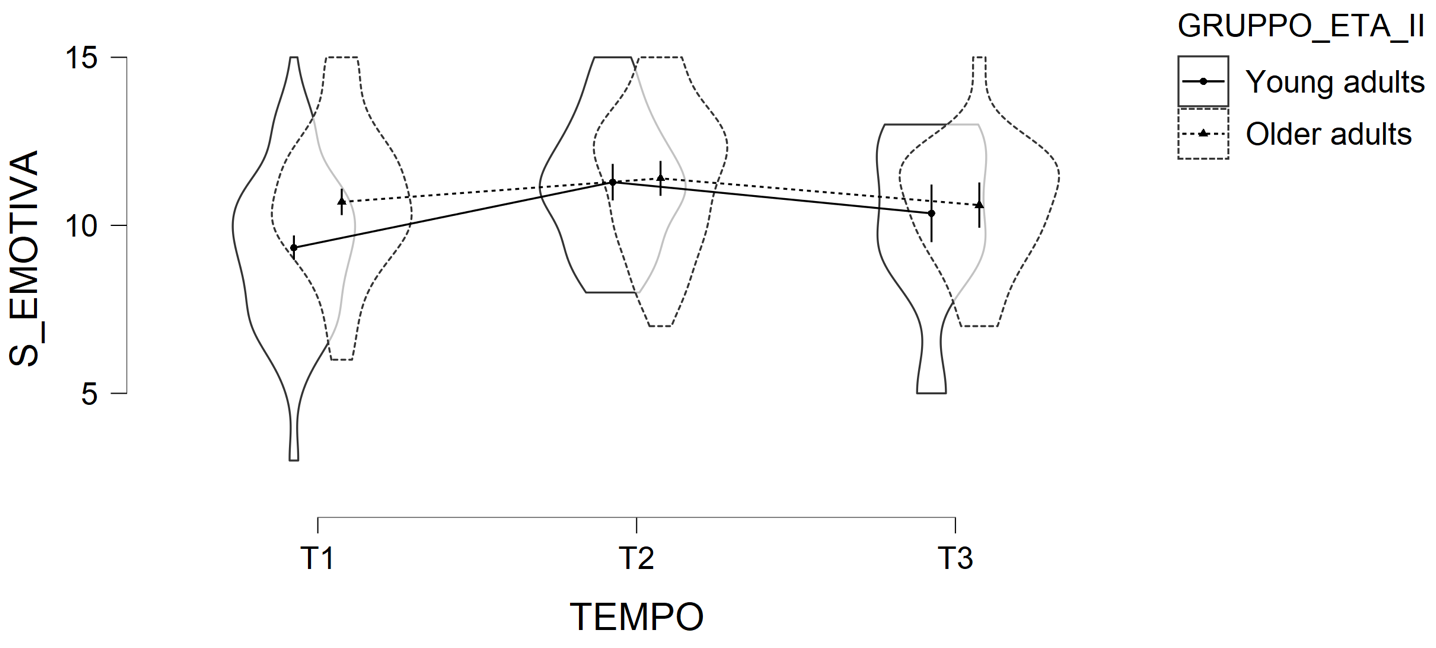

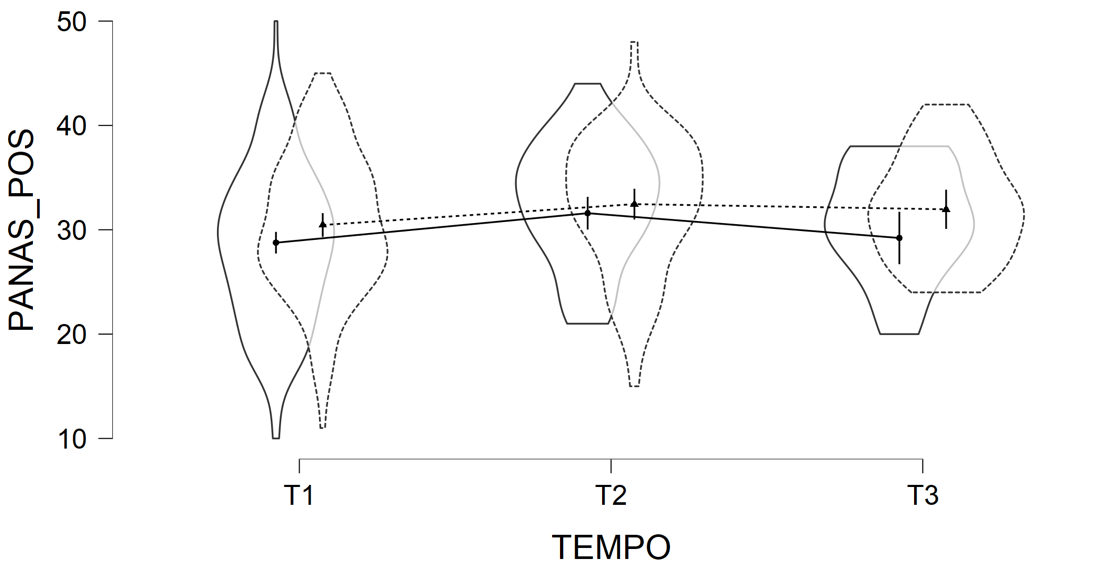


*Table S5. Model summary.*

| **Effect** | | **df** | | **χ^2^** | | ***p*** | |
| --- | --- | --- | --- | --- | --- | --- | --- |
| Mood |  | 1 |  | 79.792 |  | < .001 |  |
| Beliefs of contagion |  | 1 |  | 1.196 |  | .274 |  |
| Consequences of contagion |  | 1 |  | .189 |  | .664 |  |
| Age group |  | 1 |  | 4.017 |  | .045 |  |
| Time |  | 2 |  | 19.613 |  | < .001 |  |
| Age group X Time |  | 2 |  | 1.507 |  | .471 |  |

Results confirmed that mood was a significant covariate for the PANAS-Positive emotions. The main effect of Time showed that participants reported lower positive affect at T1 than at T2 (ß = 2.41 [1.38; 3.44] *p* < .001), but neither between T2 and T3 (ß = -1.43 [-3.01; 0.15] *p* = .230) nor between T1 and T3 (ß = 0.97 [-0.57; 2.51] *p* = .661). A main effect of Age group emerged. Older adults reported overall higher positive affect than younger adults (ß = 1.77 [0.05; 3.49] *p* < .001) when considering a longer period of time.

***PANAS-Negative emotions***

*Figure S5. Model plot for the PANAS-Negative emotions by age group and time.*


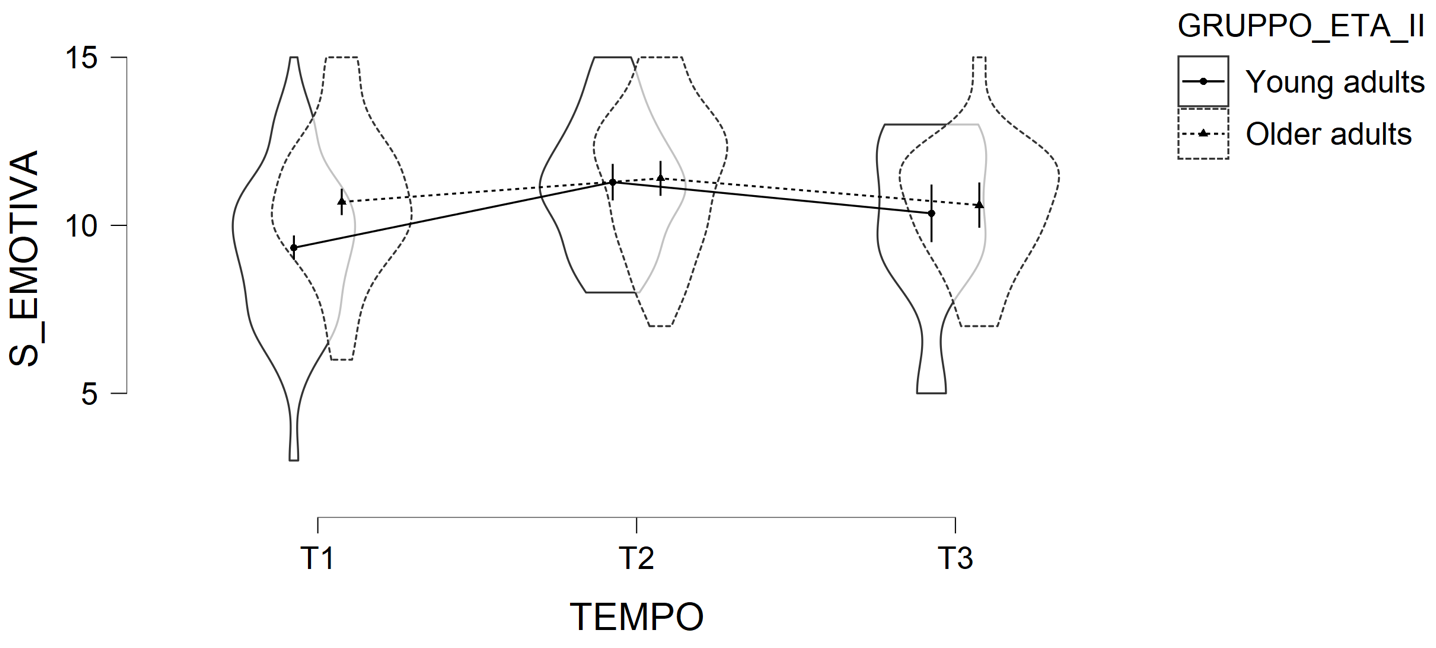

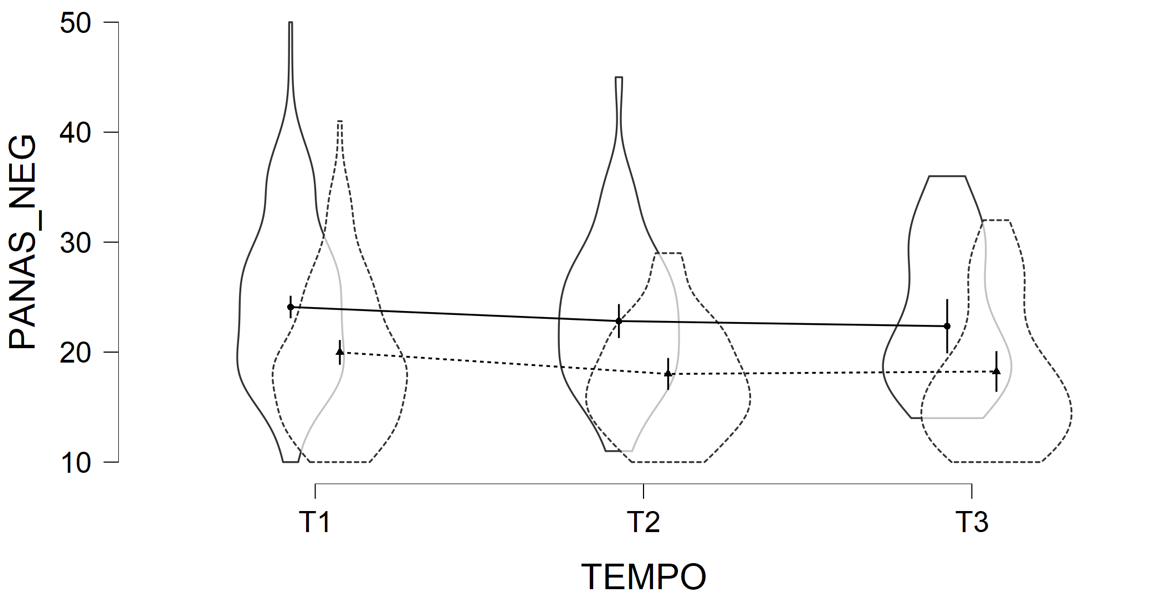


*Table S8. Model summary.*

| **Effect** | | **df** | | **χ^2^** | | ***p*** | |
| --- | --- | --- | --- | --- | --- | --- | --- |
| Mood |  | 1 |  | 80.627 |  | < .001 |  |
| Beliefs about contagion |  | 1 |  | 6.387 |  | .011 |  |
| Consequences of contagion |  | 1 |  | .153 |  | .696 |  |
| Age group |  | 1 |  | 23.871 |  | < .001 |  |
| Time |  | 2 |  | 10.831 |  | .004 |  |
| Age group X Time |  | 2 |  | .475 |  | .789 |  |

Results confirmed that mood and Beliefs about contagion were significant covariates for the PANAS-Negative emotions. Results confirmed the main effects of Age group. Young adults reported higher negative emotions than older adults (ß = 4.34 [2.64; 6.04] *p* < .001). The main effect of Time showed that participants reported higher negative emotions at T1 than at T2 (ß = -1.62 [-2.64; -0.60] *p* = .006), but neither between T2 and T3 (ß = -0.11 [-1.65; 1.42] *p* = 1.00) nor between T1 and T3 (ß = -1.73 [-3.25; -0.20] *p* = .079).

***Emotional loneliness scale***

*Figure S6. Model plot for the Emotional loneliness scale by age group and time.*


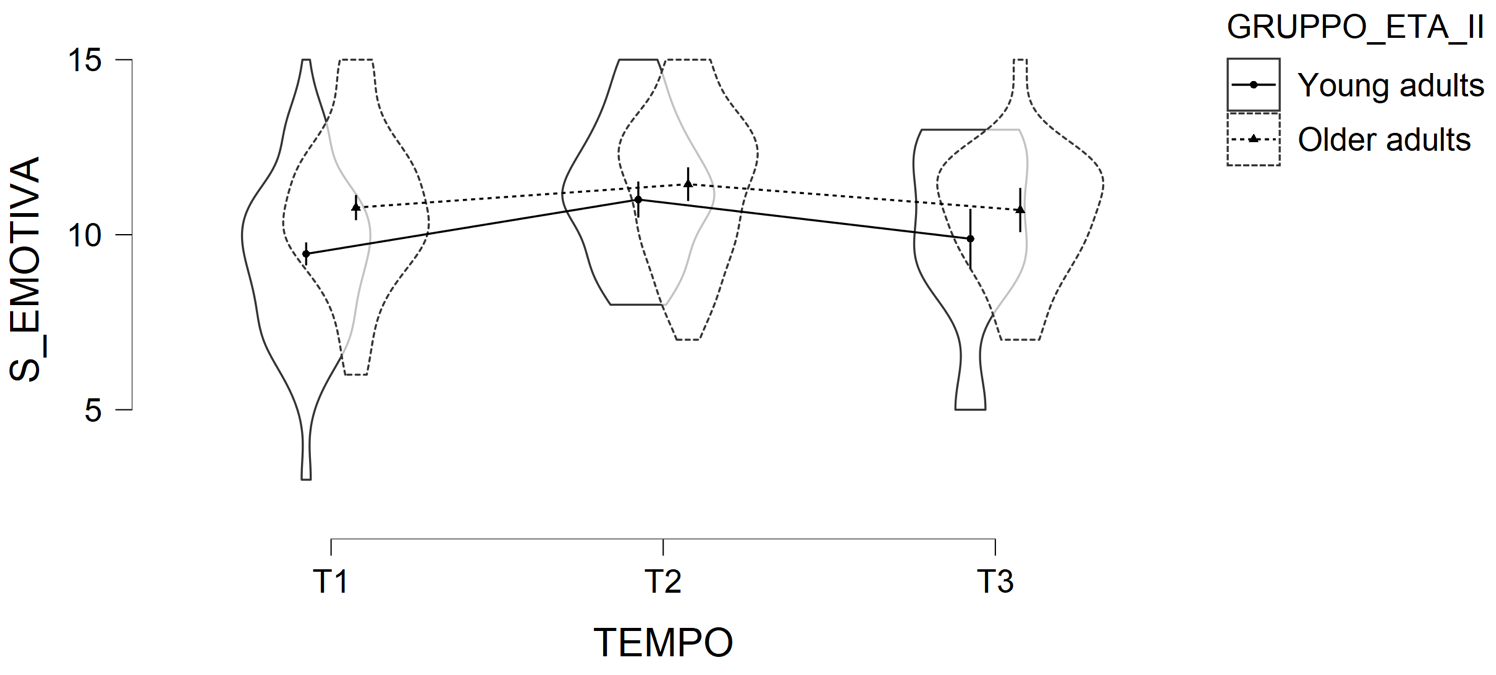

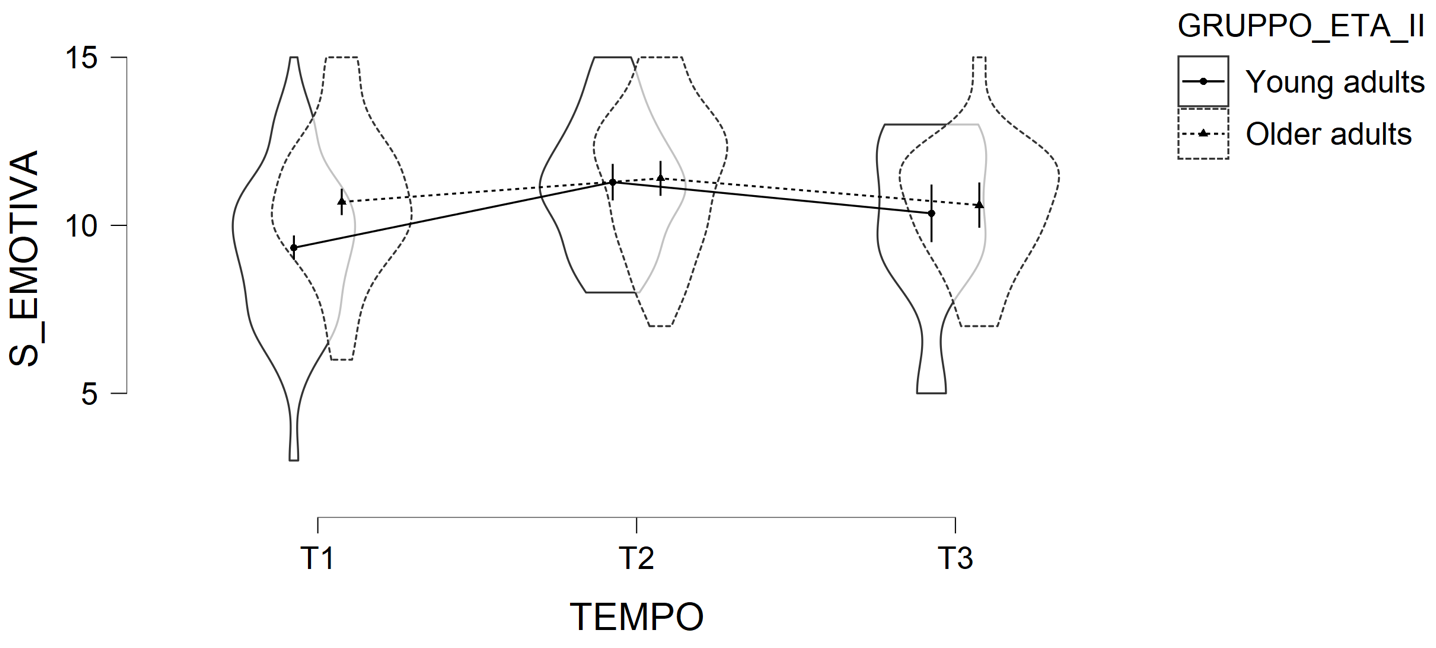


*Table S9. Model summary.*

| **Effect** | | **df** | | **χ^2^** | | ***p*** | |
| --- | --- | --- | --- | --- | --- | --- | --- |
| Mood |  | 1 |  | 89.236 |  | < .001 |  |
| Beliefs of contagion |  | 1 |  | 1.856 |  | .173 |  |
| Consequences of contagion |  | 1 |  | 0.008 |  | .927 |  |
| Age group |  | 1 |  | 9.401 |  | .002 |  |
| Time |  | 2 |  | 33.896 |  | < .001 |  |
| Age group X Time |  | 2 |  | 5.604 |  | .061 |  |

Results confirmed that mood was a significant covariate for the Emotional loneliness scale. The main effect of Age group was confirmed. Older adults overall perceived themselves as less emotionally lonelier than younger adults (ß = 0.86 [0.32; 1.40] *p* = .002). The main effect of Time showed that participants reported higher emotional loneliness at T1 than at T2 (ß = 1.11 [0.76; 1.46] *p* < .001) and felt less emotionally lonelier at T2 than at T3 (ß = -0.93 [-1.47; -0.39] *p* = .004), the latter not differing from T1 (ß = 0.18 [-0.36; 0.72] *p* = 1.00). The Age group X Time interaction was no longer significant when considering T3.

***Social loneliness scale***

*Figure S7. Model plot for the Social loneliness scale by age group and time.*


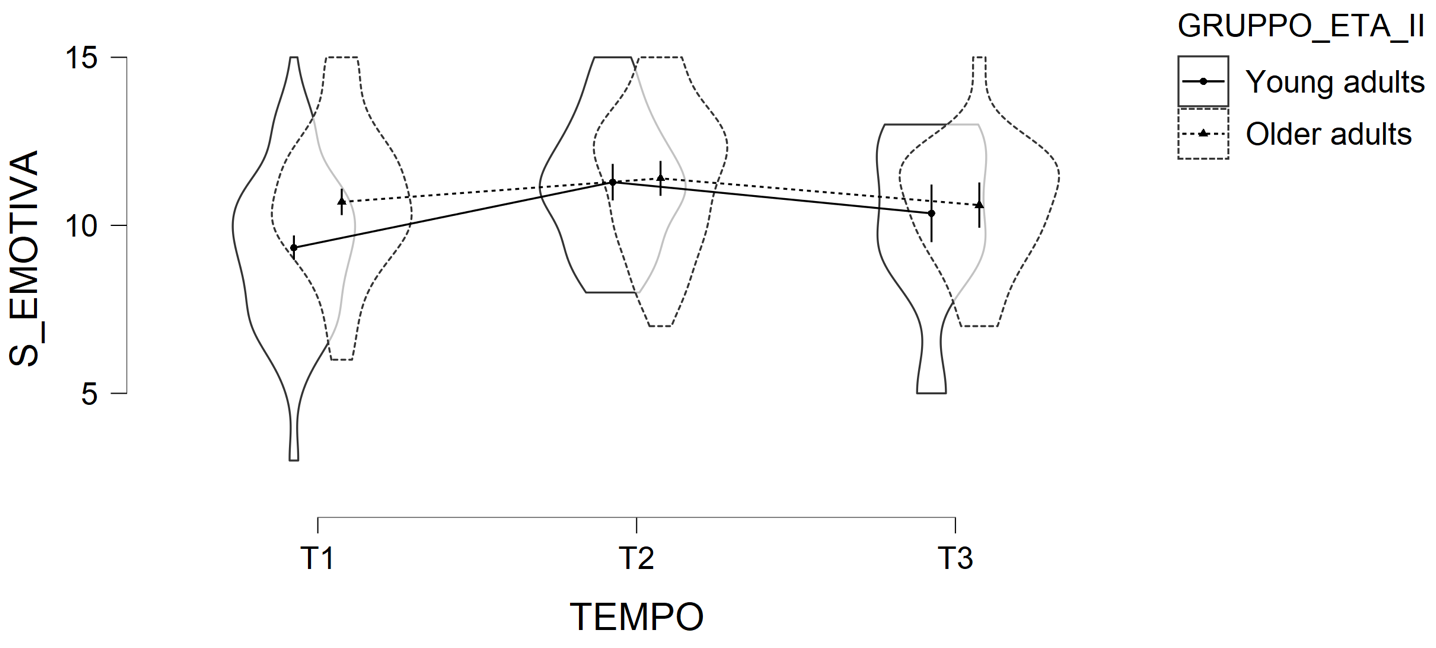

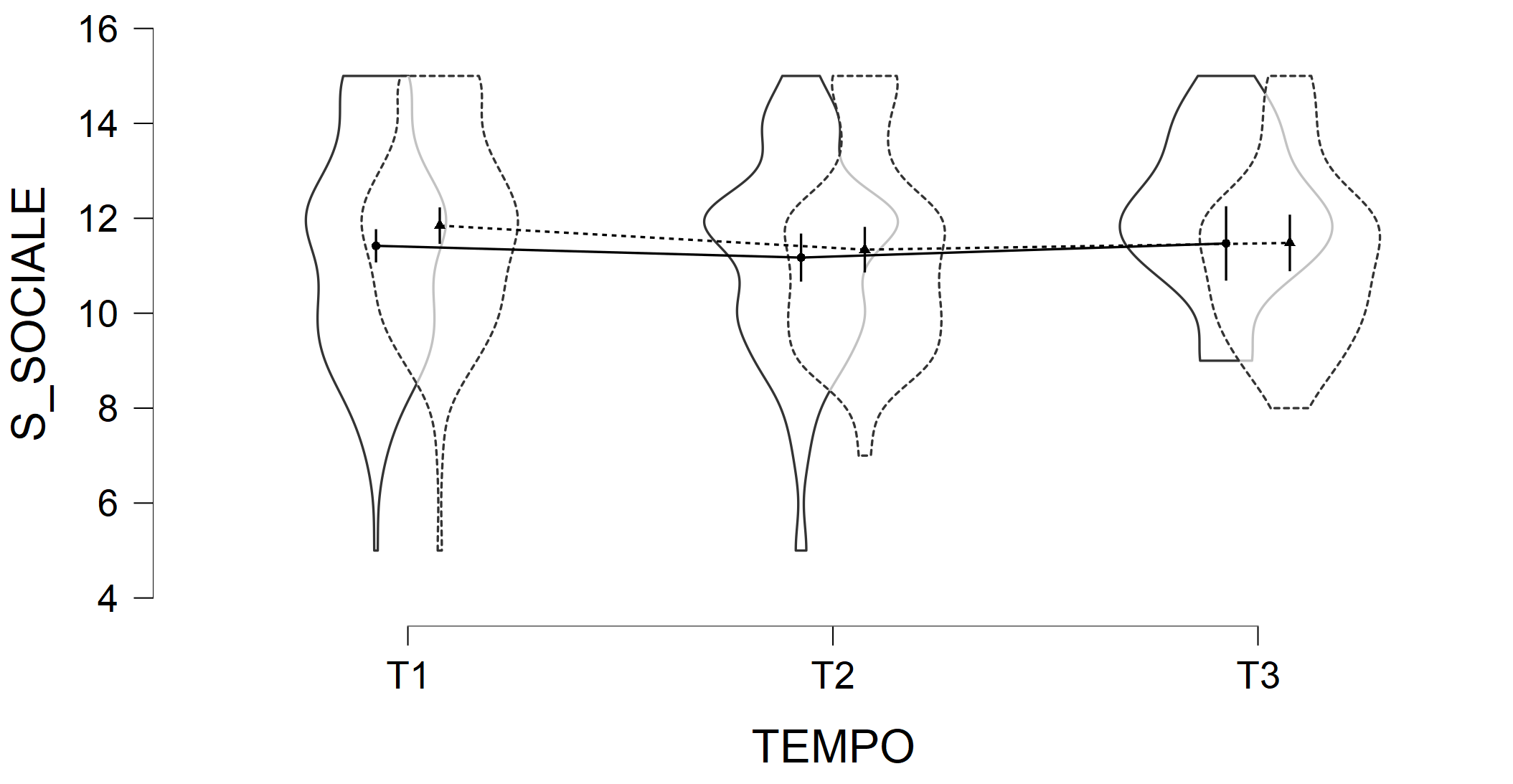


*Table S10. Model summary.*

| **Effect** | | **df** | | **χ^2^** | | ***p*** | |
| --- | --- | --- | --- | --- | --- | --- | --- |
| Mood |  | 1 |  | 17.333 |  | < .001 |  |
| Beliefs of contagion |  | 1 |  | 1.543 |  | .214 |  |
| Consequences of contagion |  | 1 |  | 1.392 |  | .238 |  |
| Age group |  | 1 |  | .465 |  | .496 |  |
| Time |  | 2 |  | 5.397 |  | .067 |  |
| Age group X Time |  | 2 |  | 1.096 |  | .578 |  |

Results confirmed that mood was a significant covariate for the Social loneliness scale. The main effect of Time was no longer significant when considering T3.

***Resilience***

*Figure S8. Model plot for the Resilience scale by age group and time.*


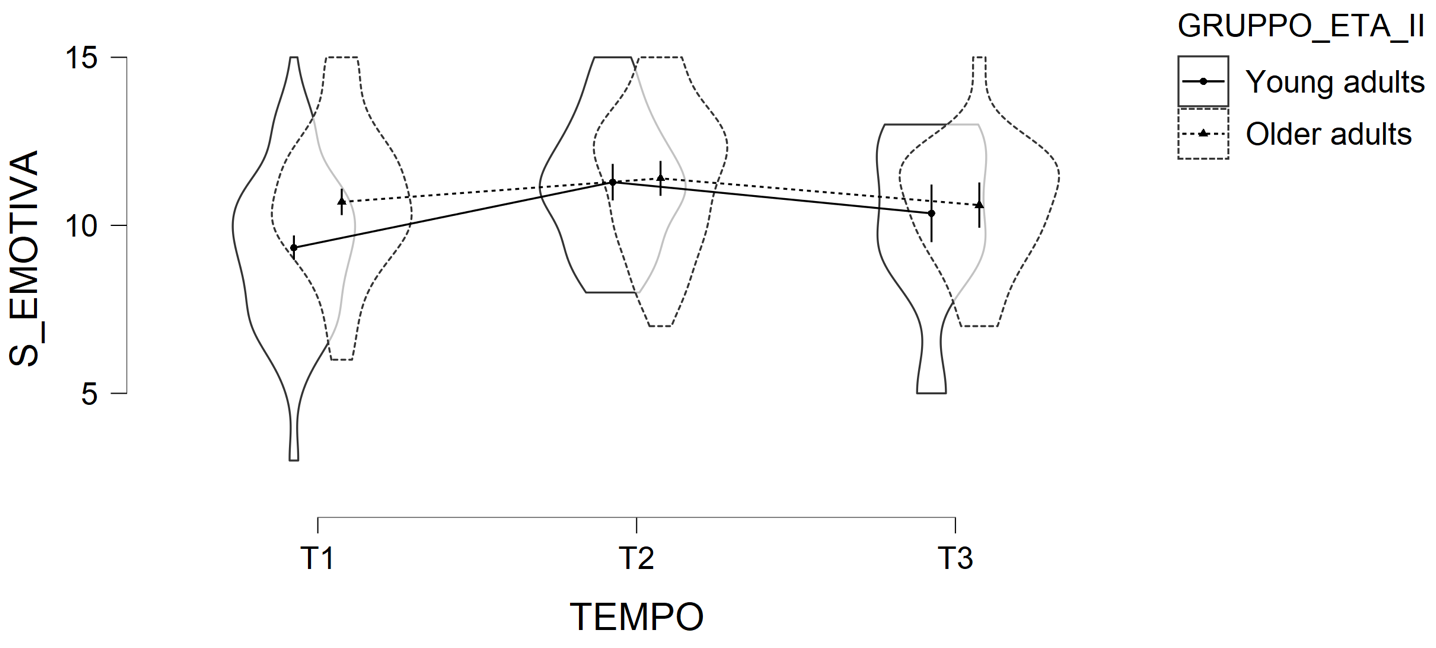

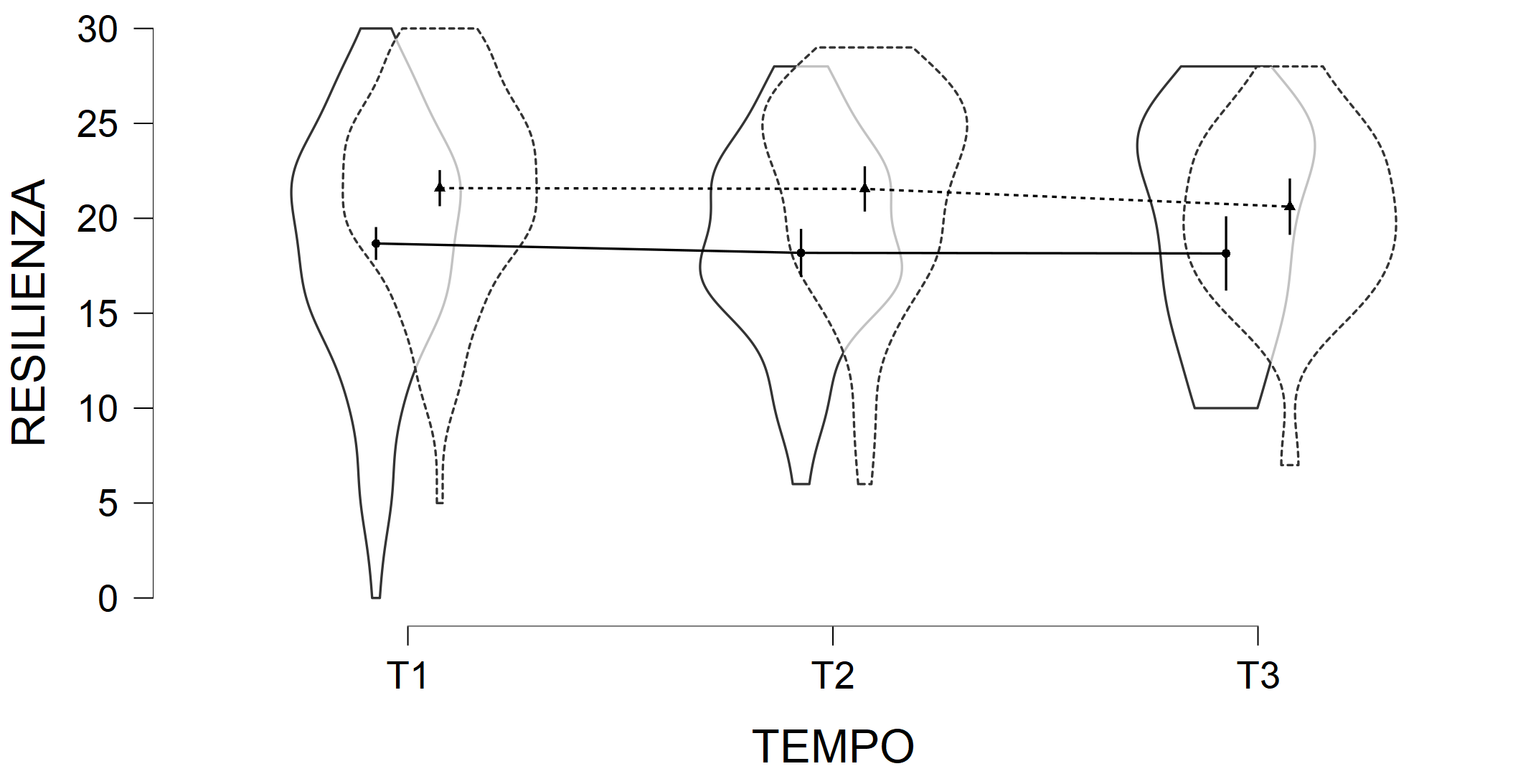


*Table S11. Model summary.*

| **Effect** | | **df** | | **χ^2^** | | ***p*** | |
| --- | --- | --- | --- | --- | --- | --- | --- |
| Mood |  | 1 |  | 64.800 |  | < .001 |  |
| Beliefs of contagion |  | 1 |  | 0.762 |  | .383 |  |
| Consequences of contagion |  | 1 |  | 0.014 |  | .907 |  |
| Age group |  | 1 |  | 15.472 |  | < .001 |  |
| Time |  | 2 |  | 1.596 |  | .450 |  |
| Age group X Time |  | 2 |  | 0.661 |  | .718 |  |

Results confirmed that mood was a significant covariate for the Resilience scale. The main effect of Age group was also confirmed. Older adults perceived themselves more resilient than younger adults (ß = 2.91 [1.48; 4.34] *p* = .001).

**Memory performance**

Linear mixed-effects models were run for all memory measures, with Age group (young adults vs. older adults) and Time (T1 vs. T2 vs. T3) as predictors first, and then also with mood (depression) and the Beliefs and Consequences of contagion scales -BCS and CCS- as covariates. Maximum likelihood estimation was used with likelihood ratio test to assess the significance of the effects.

***Backward Digit Span task***

Results from the models without covariates confirmed the main effect of Age group (χ^2^ = 35.547; *p* <.001). Younger adults outperformed older adults (ß = 2.067 [1.40; 2.734] *p* < .001). Neither the main effect of Time (χ^2^ = 4.281; *p* = .118) nor the Age group X Time interaction (χ^2^ = 0.620; *p* = .734) were significant.

*Figure S9. Model plot for the Backward Digit Span task by age group and time.*


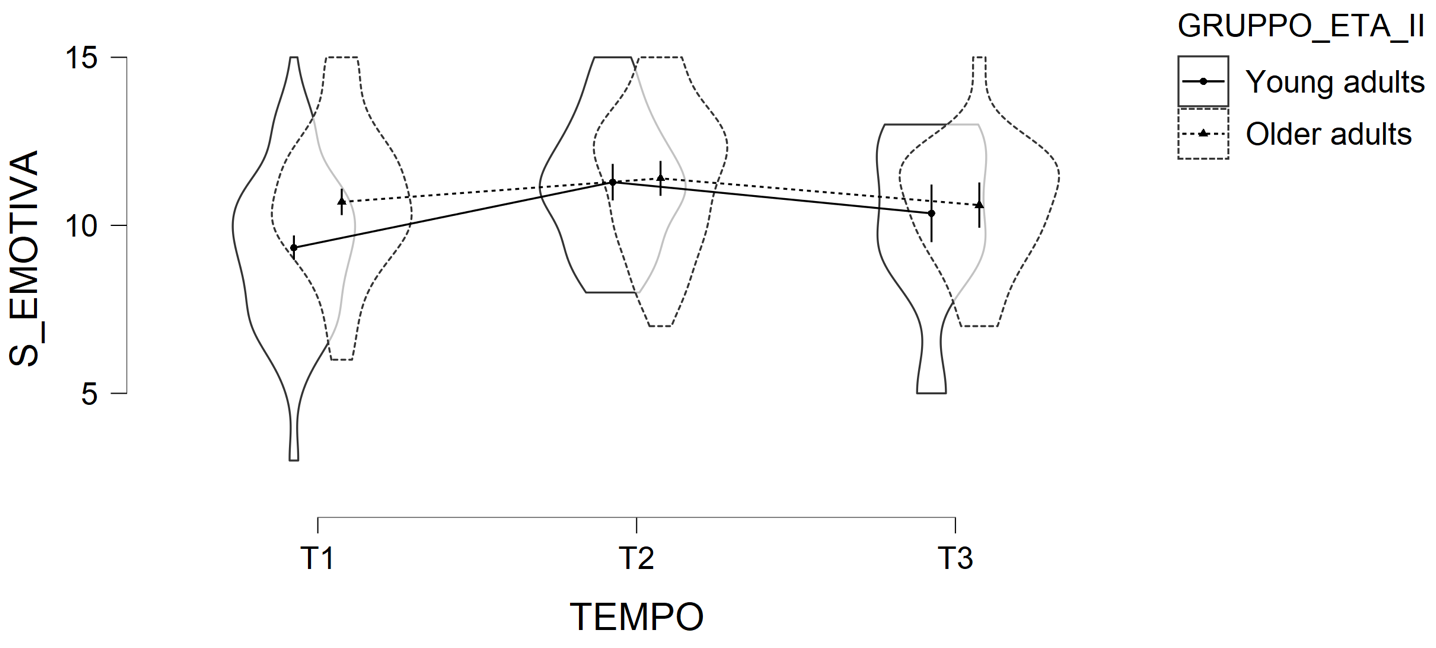

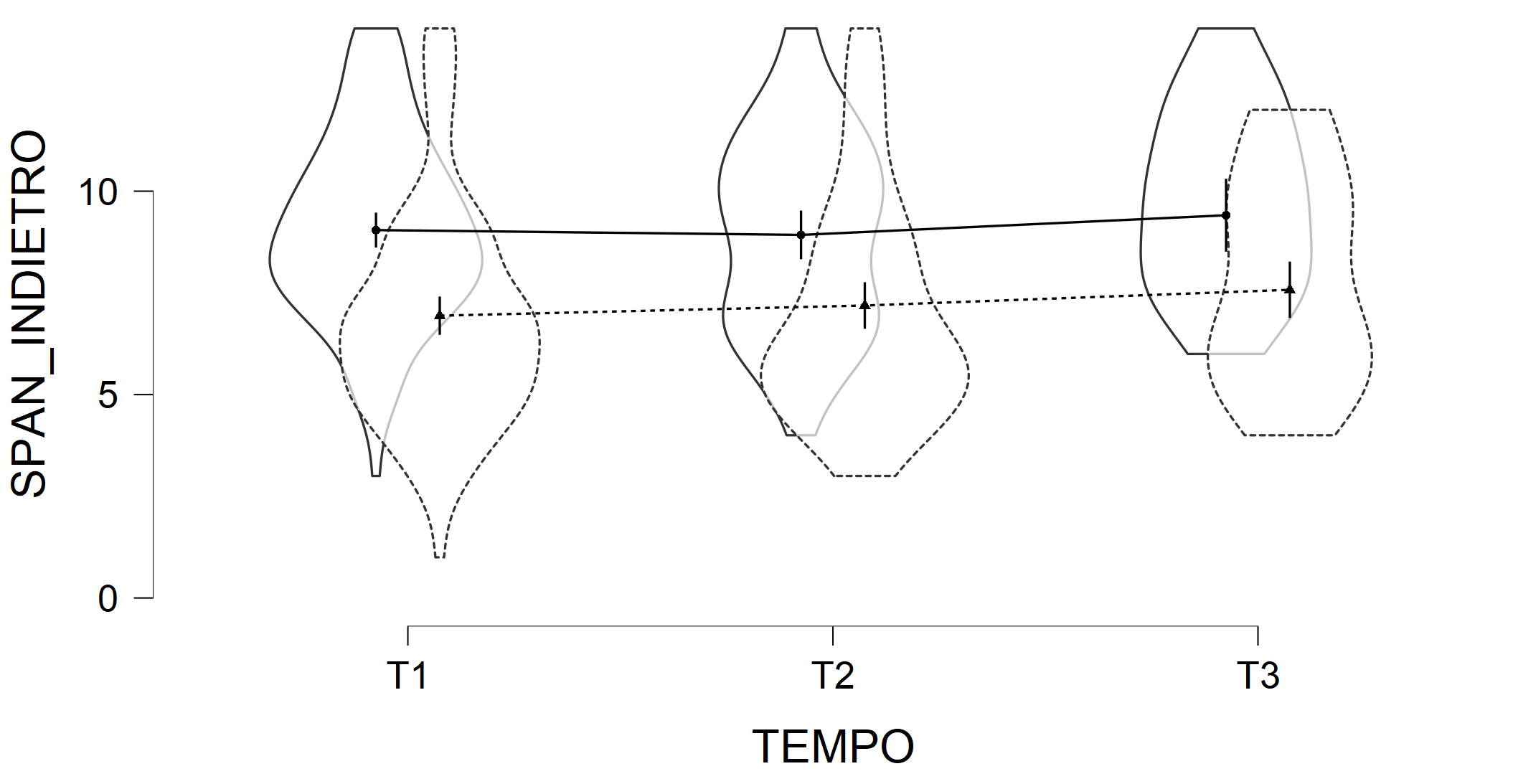


*Table S12. Model summary.*

| **Effect** | | **df** | | **χ^2^** | | ***p*** | |
| --- | --- | --- | --- | --- | --- | --- | --- |
| Mood |  | 1 |  | 3.595 |  | .058 |  |
| Beliefs of contagion |  | 1 |  | .021 |  | .884 |  |
| Consequences of contagion |  | 1 |  | 4.787 |  | .029 |  |
| Age group |  | 1 |  | 26.888 |  | < .001 |  |
| Time |  | 2 |  | 3.440 |  | .179 |  |
| Age group X Time |  | 2 |  | 1.096 |  | .578 |  |

Results from the models with covariates confirmed that the CCS was a significant covariate for the Backward Digit Span task. The main effect of Age group was also confirmed. Young adults outperformed their older counterpart (ß = 1.89 [1.21; 2.57] *p* < .001).

***Word list- immediate recall***

Results from the models without covariates confirmed the main effect of Age group (χ^2^ = 14.548; *p* <.001). Younger adults outperformed older adults (ß = 1.246 [0.613; 1.879] *p* < .001). Neither the main effect of Time (χ^2^ = 5.364; *p* = .068) nor the Age group X Time interaction (χ^2^ = 2.413; *p* = .299) were significant.

*Figure S10. Model plot for the Word list- immediate recall by age group and time.*


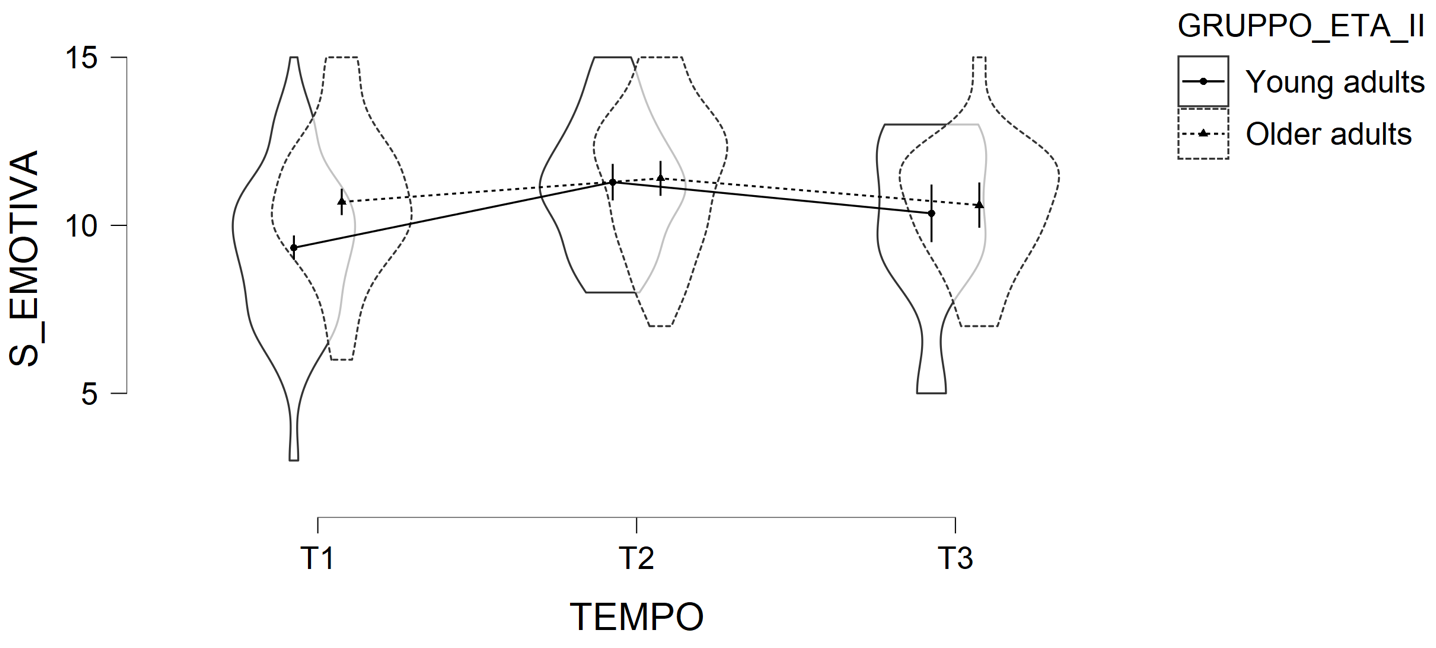

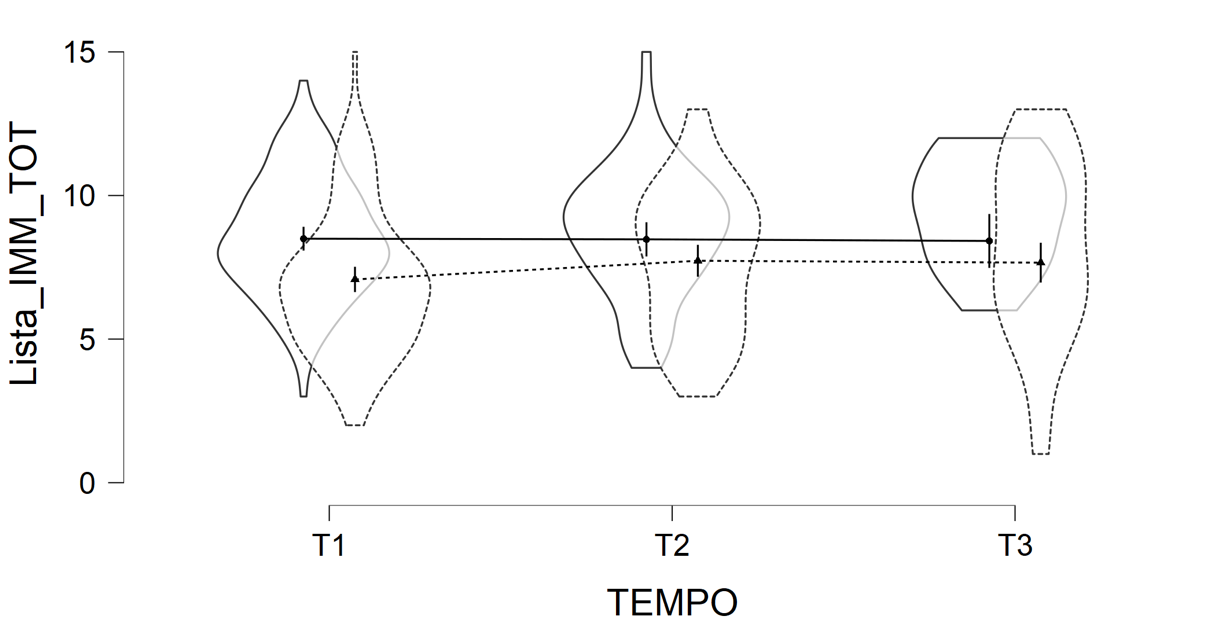


*Table S13. Model summary.*

| **Effect** | | **df** | | **χ^2^** | | ***p*** | |
| --- | --- | --- | --- | --- | --- | --- | --- |
| Mood |  | 1 |  | 5.080 |  | .024 |  |
| Beliefs about contagion |  | 1 |  | 3.473 |  | .062 |  |
| Consequences of contagion |  | 1 |  | .569 |  | .451 |  |
| Age group |  | 1 |  | 7.937 |  | .005 |  |
| Time |  | 2 |  | 2.812 |  | .245 |  |
| Age group X Time |  | 2 |  | 3.565 |  | .168 |  |

Concerning the models with covariates, mood emerged as a significant covariate for the Word list-immediate recall. The main effect of Age group was confirmed. Young adults outperformed older adults (ß = 0.97 [0.31; 1.63] *p* = .005).

***Word list- delayed recall***

Results from the models without covariates confirmed the main effect of Age group (χ^2^ = 14.587; *p* <.001). Younger adults outperformed older adults (ß = 1.542 [0.762; 2.322] *p* < .001). Neither the main effect of Time (χ^2^ = 4.735; *p* = .094) nor the Age group X Time interaction (χ^2^ = 1.834; *p* = .400) were significant.

*Figure S11. Model plot for the Word list- delayed recall by age group and time.*


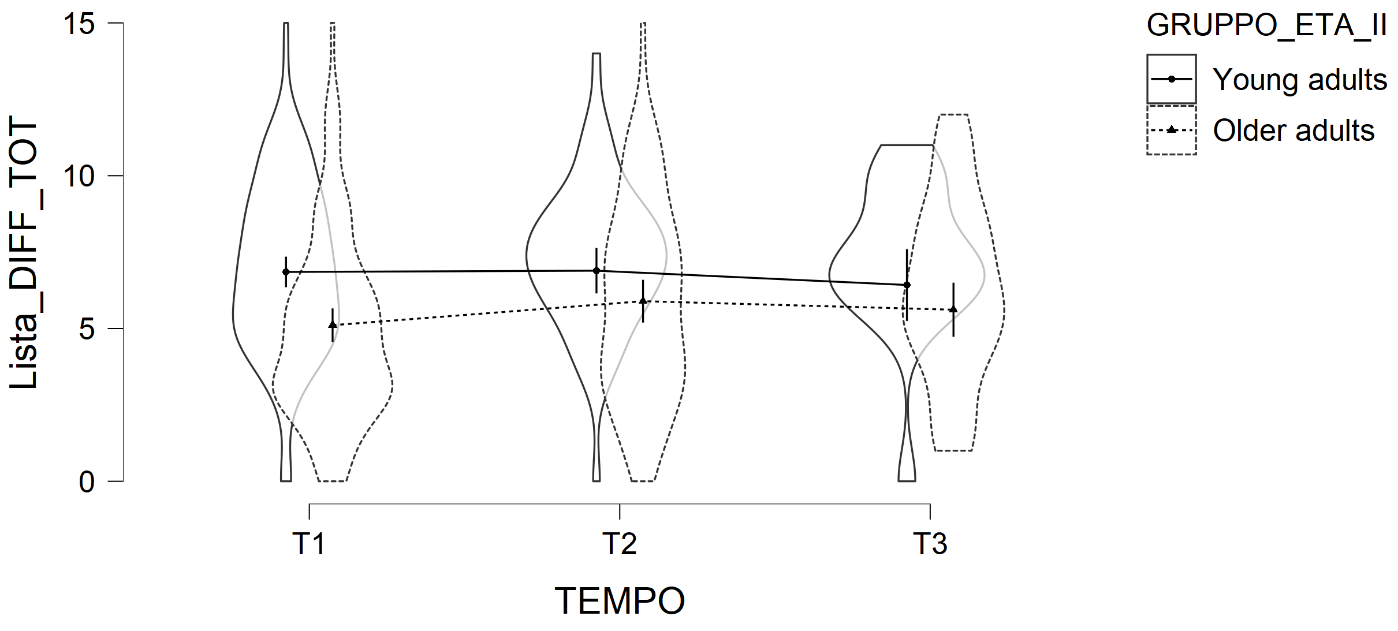

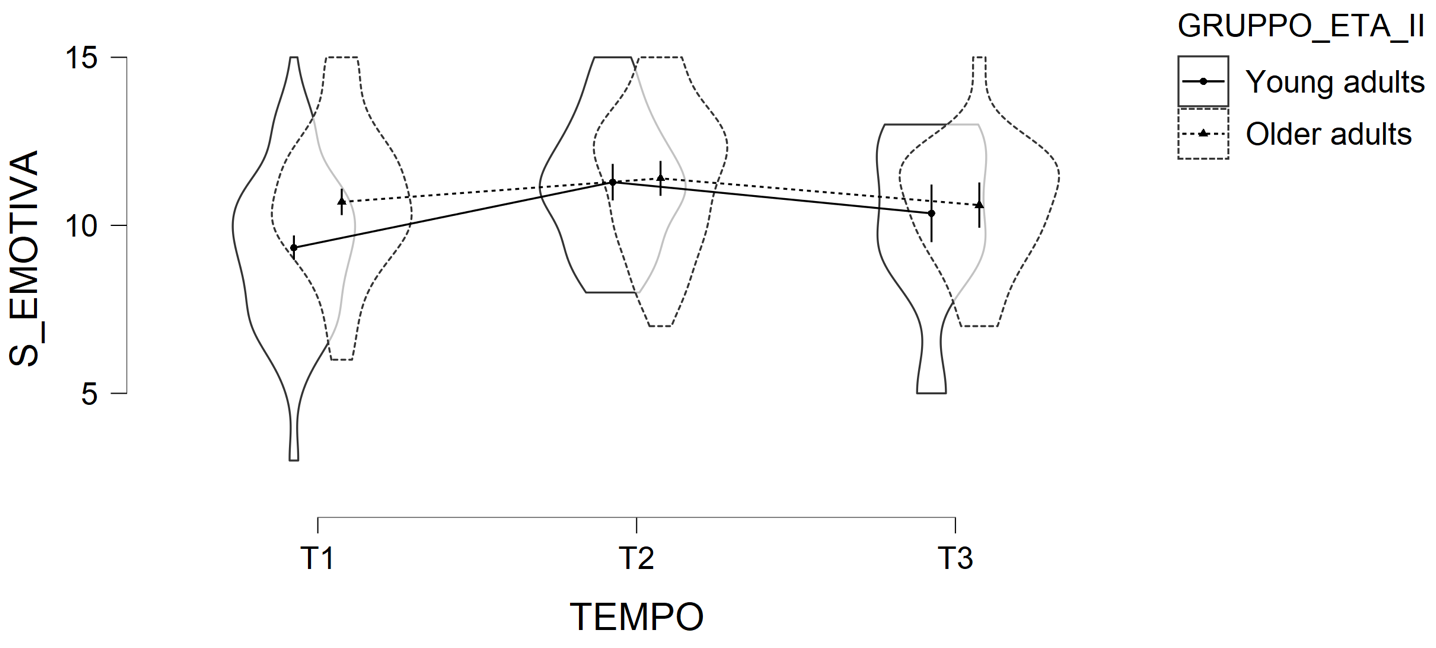


*Table S14. Model summary.*

| **Effect** | | **df** | | **χ^2^** | | ***p*** | |
| --- | --- | --- | --- | --- | --- | --- | --- |
| Mood |  | 1 |  | 4.154 |  | .042 |  |
| Beliefs about contagion |  | 1 |  | 4.712 |  | .030 |  |
| Consequences of contagion |  | 1 |  | .809 |  | .369 |  |
| Age group |  | 1 |  | 7.710 |  | .005 |  |
| Time |  | 2 |  | 2.988 |  | .225 |  |
| Age group X Time |  | 2 |  | 3.098 |  | .212 |  |

Concerning the models with covariates, results showed that mood emerged as a significant covariate, along with the BCS, for the Word list-delayed recall. The main effect of Age group was also confirmed. Younger adults outperformed older adults (ß = 1.18 [0.36; 2.65] *p* = .005).
